# Differential alterations of amygdala nuclei volumes in acutely ill patients with anorexia nervosa and their associations with leptin levels

**DOI:** 10.1017/S0033291722003609

**Published:** 2023-10

**Authors:** Marie-Louis Wronski, Daniel Geisler, Fabio Bernardoni, Maria Seidel, Klaas Bahnsen, Arne Doose, Jonas L. Steinhäuser, Franziska Gronow, Luisa V. Böldt, Franziska Plessow, Elizabeth A. Lawson, Joseph A. King, Veit Roessner, Stefan Ehrlich

**Affiliations:** 1 Translational Developmental Neuroscience Section, Division of Psychological and Social Medicine and Developmental Neurosciences, Faculty of Medicine, TU Dresden, Dresden, Germany; 2 Neuroendocrine Unit, Department of Medicine, Massachusetts General Hospital and Harvard Medical School, Boston, MA, USA; 3 Institute of Medical Psychology, Charité University Medicine Berlin, Berlin, Germany; 4 Charité University Medicine Berlin, Berlin, Germany; 5 Department of Child and Adolescent Psychiatry, Faculty of Medicine, University Hospital Carl Gustav Carus, TU Dresden, Dresden, Germany; 6 Eating Disorder Treatment and Research Center, Department of Child and Adolescent Psychiatry, Faculty of Medicine, TU Dresden, Dresden, Germany

**Keywords:** Amygdala, amygdala nuclei, anorexia nervosa, brain substructure volumes, FreeSurfer subcortical subsegmentation, structural MRI

## Abstract

**Background:**

The amygdala is a subcortical limbic structure consisting of histologically and functionally distinct subregions. New automated structural magnetic resonance imaging (MRI) segmentation tools facilitate the *in vivo* study of individual amygdala nuclei in clinical populations such as patients with anorexia nervosa (AN) who show symptoms indicative of limbic dysregulation. This study is the first to investigate amygdala nuclei volumes in AN, their relationships with leptin, a key indicator of AN-related neuroendocrine alterations, and further clinical measures.

**Methods:**

T1-weighted MRI scans were subsegmented and multi-stage quality controlled using FreeSurfer. Left/right hemispheric amygdala nuclei volumes were cross-sectionally compared between females with AN (*n* = 168, 12–29 years) and age-matched healthy females (*n* = 168) applying general linear models. Associations with plasma leptin, body mass index (BMI), illness duration, and psychiatric symptoms were analyzed via robust linear regression.

**Results:**

Globally, most amygdala nuclei volumes in both hemispheres were reduced in AN *v.* healthy control participants. Importantly, four specific nuclei (accessory basal, cortical, medial nuclei, corticoamygdaloid transition in the rostral-medial amygdala) showed greater volumetric reduction even relative to reductions of whole amygdala and total subcortical gray matter volumes, whereas basal, lateral, and paralaminar nuclei were less reduced. All rostral-medially clustered nuclei were positively associated with leptin in AN independent of BMI. Amygdala nuclei volumes were not associated with illness duration or psychiatric symptom severity in AN.

**Conclusions:**

In AN, amygdala nuclei are altered to different degrees. Severe volume loss in rostral-medially clustered nuclei, collectively involved in olfactory/food-related reward processing, may represent a structural correlate of AN-related symptoms. Hypoleptinemia might be linked to rostral-medial amygdala alterations.

## Introduction

The amygdala is a small, almond-shaped subcortical structure in the medial temporal lobe of both brain hemispheres consisting of several distinct nuclei and transition areas (LeDoux, 2007; Saygin et al., 2017). The amygdala's main functions comprise fear-/reward-associated emotional learning, regulation of aversive and appetitive behavioral responses to sensory including olfactory and gustatory stimuli, and evaluation of affective situations (Baxter & Murray, 2002; Davis & Whalen, 2001; Janak & Tye, 2015; Kim et al., 2017; Petrovich, 2011; Smitka et al., 2012). Human brain imaging studies have documented structural and functional alterations of the amygdala in various psychiatric disorders (Davis & Whalen, 2001; LeDoux, 2007; Shin & Liberzon, 2010; van Erp et al., 2016), but relatively few have focused on eating disorders (EDs) such as anorexia nervosa (AN) (Burkert, Koschutnig, Ebner, & Freidl, 2019; Donofry, Roecklein, Wildes, Miller, & Erickson, 2016; Friederich et al., 2012; Kaye, Wagner, Fudge, & Paulus, 2010; Scharner & Stengel, 2019; Seidel et al., 2018).

AN typically occurs in adolescent females and is characterized by a distorted body image, an immense fear of weight gain and a perpetual drive for weight loss, mostly by self-starvation (American Psychiatric Association, 2013). These characteristics of AN often lead to life-threatening emaciation and devastating psychological burdens (Bühren et al., 2014; Zipfel, Giel, Bulik, Hay, & Schmidt, 2015), resulting in the highest standardized mortality rate among psychiatric disorders (Arcelus, Mitchell, Wales, & Nielsen, 2011). Patients with AN often suffer from severe co-existing depressive and anxious symptoms (Fernandez-Aranda et al., 2007; Swinbourne & Touyz, 2007), display reduced fear extinction and increased fear renewal (Lambert et al., 2021), and show altered reward or punishment responses to context-specific cues (e.g. food) (Haynos, Lavender, Nelson, Crow, & Peterson, 2020). Thus, anxiety-/avoidance-based (Murray et al., 2018) as well as reward-centered (O'Hara, Campbell, & Schmidt, 2015) models of cognition and behavior in AN have been proposed. Given aforementioned functions of the amygdala as part of the limbic system (Baxter & Murray, 2002; Davis & Whalen, 2001; Janak & Tye, 2015), this brain region is assumed to play a pivotal role in AN-related psychopathology (Fuglset, Landrø, Reas, & Rø, 2016; Oldershaw, Startup, & Lavender, 2019; Scharner & Stengel, 2019).

Animal research has demonstrated high densities of diverse neurohormone, including leptin, receptors in the amygdala (Wada et al., 2014). Revealing potential associations between the amygdala and characteristic neuroendocrine alterations in humans with AN, such as suppressed leptin levels (Föcker et al., 2011; Hebebrand et al., 2022; Hebebrand, Muller, Holtkamp, & Herpertz-Dahlmann, 2007), would be crucial for understanding pathomechanisms of AN that remain largely elusive to date (King, Frank, Thompson, & Ehrlich, 2018; Scharner & Stengel, 2019; Treasure et al., 2015; Zatorre, Fields, & Johansen-Berg, 2012). In particular, hypoleptinemia is considered as a key neuroendocrine feature of AN, related to physical activity, hypothalamic–pituitary–adrenal/–gonadal/–thyroid axes activity, bone metabolism, body dissatisfaction, depressive symptom severity, disorder-specific rumination, and reward processing in these patients (Ehrlich et al., 2009a, 2009b; Fürtjes et al., 2018; Hebebrand et al., 2007, 2022; Lawson et al., 2012; Lawson & Klibanski, 2008; Schneider et al., 2009). As evident from research in rodents, leptin modulates neural circuits in the mesolimbic dopaminergic reward system including the amygdala (Opland, Leinninger, & Myers, 2010) and acts as a neuronal growth factor in the amygdala-hippocampus formation (Bouret, 2010; Ge, Fan, Yang, Cui, & Li, 2018; Lu, Kim, Frazer, & Zhang, 2006; Schepers, Gebhardt, Bracke, Eiffler, & von Bohlen und Halbach, 2020). In contrast, research on associations between leptin and brain structures/functions in humans is still sparse (Paz-Filho, 2016). However, significant leptin effects on neuronal tissue composition, functional magnetic resonance imaging (MRI) amygdala activation and (whole) amygdala volume have been described in preliminary studies in leptin-deficient patients (Frank et al., 2011, 2013; Matochik et al., 2005) and older adults (Zonneveld et al., 2021).

**Fig. 1.**
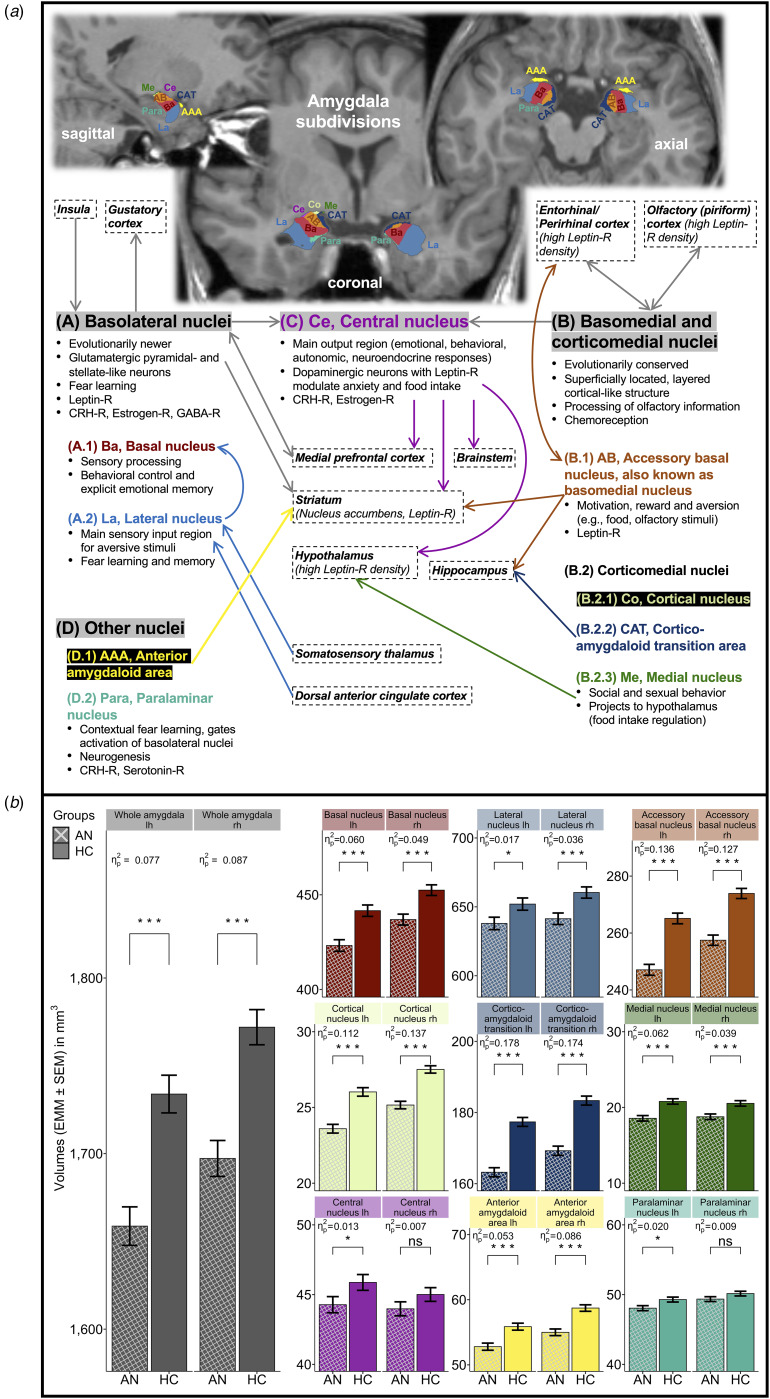
Amygdala nuclei and amygdaloid complex. (a) Two-dimensional (2D) illustration of FreeSurfer-based amygdala subsegmentation. (b) Bar graph visualization of age-, age^2^-, and eTIV-adjusted GLM0 for individual amygdala nuclei volumes in AN *v.* HC. AN, patients with acute anorexia nervosa; HC, healthy control participants; lh, left brain hemisphere; rh, right brain hemisphere; eTIV, estimated total intracranial volume; GLM, general linear model; HPA, hypothalamic–pituitary–adrenal axis; CRH, corticotropin-releasing hormone; GABA, gamma-aminobutyric acid; -R, receptor. (a) Amygdala nuclei location in axial/coronal/sagittal planes, groupings/subdivisions, functions, and connectivity (abstracted and simplified illustration). Amygdala subdivisions (A–D) are highlighted with gray background (LeDoux, 2007, 2008; McDonald, 2020; Sah et al., 2003; Saygin et al., 2011, 2017; Watson et al., 2012). Brain regions in boxes with dashed outline refer to important input or output regions. Arrows indicate predominantly unidirectional or reciprocal connectivity (gray arrows, joined projections to/from all nuclei within an amygdala subdivision; arrows colored according to nuclei labels, projections to/from individual amygdala nuclei). 2D FreeView-snapshots of a T1-weighted sMRI scan after FreeSurfer version 7.1.1-based amygdala subsegmentation, mapped onto a preprocessed and normalized T1-weighted brain image of a typical patient with acute AN in the study sample. (b) Bar graphs with error bars for study groups AN (*n* = 168) and age-matched HC (*n* = 168) displaying adjusted means (EMM, mm^3^) ± standard error of the mean (s.e.m.) of individual whole amygdala and amygdala nuclei volumes in separate brain hemispheres (color of bars matches color of nuclei labels in panel a). Model estimates were obtained with GLM0 [performed separately for each amygdala (sub-)region, computed as *F* test: dfs = 1, 331] covarying for age at date of research (linear and quadratic orthogonal polynomials) and eTIV (covariates were grand mean-centered). FDR-q, *p* values were multiple testing adjusted using false-discovery rate (Benjamini & Hochberg, 1995) across all amygdala nuclei (whole amygdala adjusted separately using FDR). Significance levels for volume differences between study groups are stated as: ***q < 0.001; *q < 0.05; ns, non-significant. Effect size statistics are provided as partial *η*^2^ (Cohen, 1988).

Previous structural MRI (sMRI) studies found global and regionally unspecific brain mass reductions in AN including cortical thickness and subcortical gray matter (GM) volumes such as the amygdala as a whole (Bahnsen et al., 2022; Bernardoni et al., 2016; Burkert et al., 2019; Eynde et al., 2012; Friederich et al., 2012; King et al., 2015; Monzon et al., 2017; Seitz et al., 2014; Seitz, Herpertz-Dahlmann, & Konrad, 2016; Su et al., 2021; Titova, Hjorth, Schiöth, & Brooks, 2013; Walton et al., 2022). Accumulating evidence from rodent models and post-mortem human brain samples points toward the phylogenetic, histological, and functional heterogeneity of the amygdala or, more precisely, the amygdaloid complex (Sah, Faber, Lopez De Armentia, & Power, 2003; Saygin et al., 2017; Saygin, Osher, Augustinack, Fischl, & Gabrieli, 2011). Hence, the amygdaloid complex is often subdivided into basolateral, baso-/corticomedial, central, and other nuclei based on differing neuronal cell types, neurotransmitter profiles, and intra-/extra-amygdaloid structural and functional connectivity (Fig. 1a): for instance, evolutionarily newer basolateral nuclei consist of glutamatergic neurons and function as input gateways for emotionally significant (e.g. fear-/stress-induced or rewarding) stimuli into the amygdaloid complex, whereas central nucleus with dopaminergic neurons regulates autonomic, neuroendocrine, and behavioral responses to these stimuli. Evolutionarily conserved accessory basal, cortical, medial nuclei, and corticoamygdaloid transition communicate with the olfactory cortex and exercise emotional control over food intake (LeDoux, 2007, 2008; Sah et al., 2003; Saygin et al., 2011, 2017). These findings encourage the consideration of amygdala substructures also in translational and clinical research (LeDoux, 2007) as in the current study on AN. Potential substructural amygdala alterations may differ from generic GM alterations in AN, i.e. be subregion-specific and, thus, of putative clinical importance. Until recently, substructure-level investigations of the amygdaloid complex have not been feasible in larger human samples applying standard sMRI techniques (Saygin et al., 2011). Newly developed automated tools for amygdala subsegmentation into distinct nuclei integrated in FreeSurfer (Saygin et al., 2017) have prompted pilot studies on amygdala nuclei alterations in psychiatric populations (Morey et al., 2020; Zheng et al., 2019) but not yet in AN or other EDs.

Here we investigated for the first time the volumes of individual nuclei and transition areas of the amygdaloid complex in a large sample of acutely underweight females with AN in comparison with age-matched healthy females. We applied FreeSurfer-based amygdala subsegmentation (Fischl, 2012; Saygin et al., 2017) and multi-stage sMRI quality assessment. Given evidence for widespread (sub-)cortical neuroanatomical alterations in AN (King et al., 2018), we expected that amygdala nuclei volumes would also be reduced in the AN group. Importantly, however, we strived to elucidate differences in the relative severity of amygdala nuclei alterations to unveil potential significant effects beyond whole amygdala and other subcortical GM volume alterations. Specifically, motivated by anxiety-/reward-based models of AN (Murray et al., 2018; O'Hara et al., 2015) and the above-noted functional amygdala subdivisions, we hypothesized that volumetric reductions would be most prominent in basolateral and baso-/corticomedial amygdala subdivisions predominantly involved in fear and reward processing (Baxter & Murray, 2002; Davis & Whalen, 2001; Janak & Tye, 2015; LeDoux, 2007; Sah et al., 2003). Finally, we explored the clinical relevance of amygdala nuclei alterations in AN by investigating associations with leptin levels serving as an indicator of AN-related neuroendocrine alterations (Hebebrand et al., 2007 2022), degree of underweight, illness duration, and ED-specific, depressive, anxious, and general psychiatric symptom levels.

## Methods

### Participants

Female patients with acute AN were admitted to ED treatment programs at a child and adolescent psychiatry or psychosomatic medicine department of a tertiary care university hospital and underwent MRI within 96 h after beginning nutritional rehabilitation. Current AN, according to DSM-5 criteria, was diagnosed using a modified version of the expert form of the Structured Interview for Anorexia and Bulimia Nervosa (SIAB-EX) (Fichter & Quadflieg, 1999) and required a body mass index (BMI) <10th age percentile (if younger than 18 years) or <17.5 kg/m^2^ (if 18 years and older). Female healthy control participants (HC) were recruited through advertisements among middle school, high school, and university students, selectively to match AN for age. HC had to be of normal weight, eumenorrheic, mentally healthy, and show normal eating behavior, assessed via SIAB-EX (Fichter & Quadflieg, 1999). HC were excluded if they had any history of a psychiatric illness, a lifetime BMI <10th age percentile (if younger than 18 years) or <17.5 kg/m^2^ (if 18 years and older). Regarding ethnicity, all study participants identified as ‘European’. See Table 1 and online Supplementary Material (SM) 1.1–1.2 for further details about the study sample [socioeconomic status (SES), exclusion criteria for all participants and potential confounders such as cigarette smoking; AN subtype, duration of illness (DOI), co-existing psychiatric diagnoses, and antidepressant medication in AN (selective serotonin reuptake inhibitors or mirtazapine in *n* = 5 patients)]. The study was approved by the Institutional Review Board of the TU Dresden and carried out in accordance with the Declaration of Helsinki of 1975, as revised in 2008. All study participants (and their legal guardians if underage) gave written informed consent or assent (if underage).

**Table 1.** Demographic variables and clinical measures

	*n*	Sample mean ± s.d. Median ± IQR		Analyses			
	AN/HC	AN	HC	*t* *W*	df	*p*	Cohen's *d* Wilcoxon's *r*
Demographics							
Age (years)	168/168	16.42 ± 3.04	16.61 ± 2.97	0.57	334	0.569	0.062
SES^a^	140/123	3.50 ± 2.00^b^	4.00 ± 1.50^b^	9832^b^	n/a^b^	0.043^b^	0.125^b^
IQ	154/167	111.91 ± 12.02	112.84 ± 10.19	0.74	319	0.460	0.083
Nutritional status							
BMI (kg/m^2^)	168/168	14.67 ± 1.41	20.80 ± 2.24	30.04	334	<0.001	3.277
BMI-SDS	168/168	−3.27 ± 1.25	−0.01 ± 0.67	29.64	334	<0.001	3.234
BMI_min_ (kg/m^2^)	163/138	14.31 ± 1.40	19.82 ± 1.94	27.83	299	<0.001	3.304
Hormone parameter							
Leptin (μg/L)	142/156	1.53 ± 2.28	12.76 ± 9.02	n/a	n/a	n/a	n/a
Log_10_-leptin	142/156	−0.36 ± 0.85	1.01 ± 0.31	18.74	296	<0.001	2.187
Brain segmentation volumes							
eTIV (mm^3^)	168/168	1 495 264 ± 122 718	1 512 042 ± 105 759	1.34	334	0.180	0.146
Subcortical GM volume (mm^3^)	168/168	57 929 ± 4624	61 135 ± 4123	8.89	331	<0.001	0.977
Psychiatric symptom measures							
EDI-2 core	164/161	26.02 ± 7.02	14.70 ± 4.45	17.40	323	<0.001	1.923
BDI-II total	166/160	23.23 ± 11.02	4.56 ± 4.50	20.17	324	<0.001	2.205
STAI(K) trait anxiety	141/149	48.02 ± 12.93	33.13 ± 7.40	11.95	288	<0.001	1.424
SCL-90-R GSI	165/160	1.00 ± 0.63	0.27 ± 0.28	13.55	323	<0.001	1.488

AN, patients with acute anorexia nervosa; HC, healthy control participants; SES, socioeconomic status; IQ, intelligence quotient; BMI, body mass index; BMI-SDS, body mass index standard deviation score; BMI_min_, minimum lifetime BMI; log_10_-leptin, logarithmically transformed (base 10) leptin concentration; eTIV, estimated total intracranial volume; subcortical GM volume, total subcortical gray matter volume; EDI-2 core, averaged score comprising the core subscales ‘drive for thinness’, ‘body dissatisfaction’, and ‘bulimia’ of Eating Disorder Inventory-2; BDI-II, Beck Depression Inventory-II; STAI, State-Trait Anxiety Inventory (for participants aged ⩾15 years); STAI(K), State-Trait Anxiety Inventory for Children (for participants aged <15 years); SCL-90-R GSI, Global Severity Index of the Symptom Checklist-90-Revised.

Number of participants and mean value ± standard deviation (s.d.) for each variable and study group (AN, HC) are shown. Group differences were tested using two-sample *t* tests (age-, age^2^-, and eTIV-adjusted GLM for total subcortical GM volume). As test statistics, *t* value (absolute value), degrees of freedom (df), *p* value, and effect size estimate Cohen's *d* (Cohen, 1988) are stated. Censored likelihood multiple imputation (Boss et al., 2019) was applied for left-censored leptin values in AN below the lower limit of detection of the leptin assay (LOD = 0.20 μg/L). General brain segmentation measures eTIV and total subcortical GM volume were extracted from FreeSurfer's segmentation statistics (Fischl, 2012). In AN, the mean ± s.d. age at first onset of AN was 14.5 ± 2.8 years (assessed in *n* = 163) and the mean ± s.d. duration of the current AN episode (DOI) was 14.1 ± 18.3 months (*n* = 164). AN subtype was determined via SIAB-EX: 142 AN (84.52%) were restrictive and 22 (13.10%) were binge-purge [subtype not assessed in *n* = 4 (2.38%)]. Of the patients with AN, 26 had one or more co-existing psychiatric conditions: 12 had a depressive disorder, 11 an anxiety disorder, 6 an obsessive-compulsive disorder, 1 a post-traumatic stress disorder, 1 an adjustment disorder, 2 a personality disorder, 1 a developmental disorder, 1 Tourette syndrome, and 1 a somatization disorder. Selective serotonin reuptake inhibitors were taken by 4 AN and mirtazapine by 1 AN within the last 6 months before study participation. None of the HC participants had any psychiatric diagnosis currently or in the past or any psychoactive medication. All study participants were female and identified as ‘European’.

a SES was determined according to the parental (household) educational level/occupation group [range: 0 (lowest), leaving school without graduation – 5 (highest), graduating from university] (Patrick et al., 2004), given most study participants were adolescent, current students at school, university, or professional training institutions (AN: 83.33%, HC: 77.38%) and still lived with their parents or guardians (AN: 85.71%, HC: 68.45%). See online SM 1.2 for further details.

b Median ± interquartile range (IQR) are shown for SES (ordinal scale), and group differences in SES were tested using Wilcoxon rank-sum test with continuity correction (*W*, *p* value, and effect size estimate Wilcoxon's *r* are stated as test statistics).

The initial study sample was subjected to quality control (QC) of sMRI scans; participants with misapplied general (sub-)cortical segmentation and/or amygdala subsegmentation were discarded (online SM 1.4–1.5, Table S1). To optimize group comparisons, HC were age-matched to AN via optimal pair matching pursuing a minimized sum of absolute pairwise distances in the matched sample (Hansen & Klopfer, 2006). The difference in age means between matched groups was 0.2 years (maximum age distance among AN–HC pairs was 0.6 years). The final study sample consisted of 336 female volunteers: 168 AN (aged 12–29 years) and 168 age-matched HC (aged 12–29 years).

### Clinical measures

ED-specific symptoms were assessed with Eating Disorder Inventory-2 (EDI-2) (Paul & Thiel, 2005), depressive symptoms with Beck Depression Inventory-II (BDI-II) (Hautzinger, Keller, & Kühner, 2009), trait anxiety symptoms with State-Trait Anxiety Inventory [STAI(K)-trait] (Spielberger, 2010), and general psychiatric symptoms with Symptom Checklist-90-Revised (SCL-90-R Global-Severity-Index/GSI) (Franke, 2002). In line with literature (Hellerhoff et al., 2021; Monteleone et al., 2019), a summary score representing ‘core’ ED symptoms was calculated by averaging EDI-2 subscales ‘drive for thinness’, ‘body dissatisfaction’, and ‘bulimia’ (online SM 1.2). BMI standard deviation score (BMI-SDS, age-/gender-adjusted) was used for analyses (Hemmelmann, Brose, Vens, Hebebrand, & Ziegler, 2010; Kromeyer-Hauschild et al., 2001). Study data were collected and managed using a secure, web-based electronic data capture tool (REDCap) (Harris et al., 2009).

For leptin measurements, fasting venous blood was collected into EDTA vacutainer tubes at 7–9 a.m., for AN within 96 h after treatment initiation. Plasma leptin was measured using a commercially available enzyme-linked immunosorbent assay (BioVendor, Brno, Czech Republic) with intra-/inter-assay variation coefficients <6%. Leptin values were logarithmically transformed (log_10_-leptin) to achieve normality (Haas et al., 2005). Plasma samples were available from 142 AN and 156 HC. Non-detectable leptin concentrations below the lower limit of detection of the leptin assay (LOD = 0.20 μg/L) occurred in 39 of 142 AN (27.46%) and were subsequently imputed using censored likelihood multiple imputation to preserve their natural variability [CLMI (Boss et al., 2019), online SM 1.3; see online SM 2.4 for confirmatory analysis including only leptin levels ⩾LOD in AN]. Leptin levels <LOD did not occur in HC. Missing/unavailable leptin values were not imputed.

### MRI data acquisition and processing

All participants underwent MRI between 8 a.m. and 9 a.m. following an overnight fast. High-resolution three-dimensional (3D) T1-weighted structural scans were acquired on a 3.0T scanner (Magnetom Trio, Siemens, Erlangen, Germany) using a MP-RAGE sequence with the following parameters: 176 sagittal slices (thickness = 1 mm, no gap), TR = 1900 ms, TE = 2.26 ms, flip angle = 9°, voxel size = 1.0 × 1.0 × 1.0 mm^3^, FoV = 256 × 224 mm^2^, bandwidth = 200 Hz/pixel. MRI data were processed in a fully automated manner (online SM 1.4) with FreeSurfer (http://surfer.nmr.mgh.harvard.edu, version 7.1.1) to achieve cortical surface reconstruction and volumetric brain segmentation including subcortical processing streams (Fischl, 2012).

The automated amygdala subsegmentation into nuclei was performed using FreeSurfer functionality for combined amygdala/hippocampus subsegmentation, based on a Bayesian probabilistic atlas to assign an anatomical label to each voxel (Saygin et al., 2017). The atlas was created from high-resolution *ex-vivo* MRI data (≈0.1 mm isotropic at 7 T, manually segmented post-mortem human brain samples) and *in-vivo* training MRI data (Saygin et al., 2017). Ten amygdala (sub-)region volumes were generated and analyzed separately for left (lh) and right (rh) brain hemispheres: whole amygdala, accessory basal, basal, central, cortical, lateral, medial, and paralaminar nuclei, anterior amygdaloid area, and corticoamygdaloid transition.

### Quality control

There are no established QC standards for amygdala subregions to date (Sämann et al., 2020). Therefore, we developed a visual and partly outlier-guided multi-stage QC procedure for combined amygdala/hippocampus subsegmentations in line with recently published recommendations for hippocampal subfield QC by the ENIGMA consortium (Sämann et al., 2020) and under expert consultation with two independent ENIGMA representatives (http://enigma.ini.usc.edu/). Briefly, our QC involved: (1) *a-priori* exclusion of participants with low scan quality (contrast-/signal-to-noise ratios) or insufficient ratings in general (sub-)cortical QC (online SM 1.4), (2) snapshot-based visual QC of amygdala/hippocampus subsegmentations of all participants after *a-priori* exclusions, and (3) dynamic visual inspection of amygdala/hippocampus subsegmentations with statistical outliers using FreeSurfer's FreeView tool (based on group-wise outlier detection via combined volume and bilateral symmetry criteria). Visual QC was manually conducted by two trained raters with substantial interrater reliability (*κ* = 0.76, online SM 1.4). Participants with misapplied amygdala/hippocampus subsegmentation were excluded [3.35% overall which is similar to previous hippocampal subfield studies (3.50%) (Sämann et al., 2020), online SM 1.4, Fig. S1, Table S1].

### Statistical analyses

**Fig. 2.**
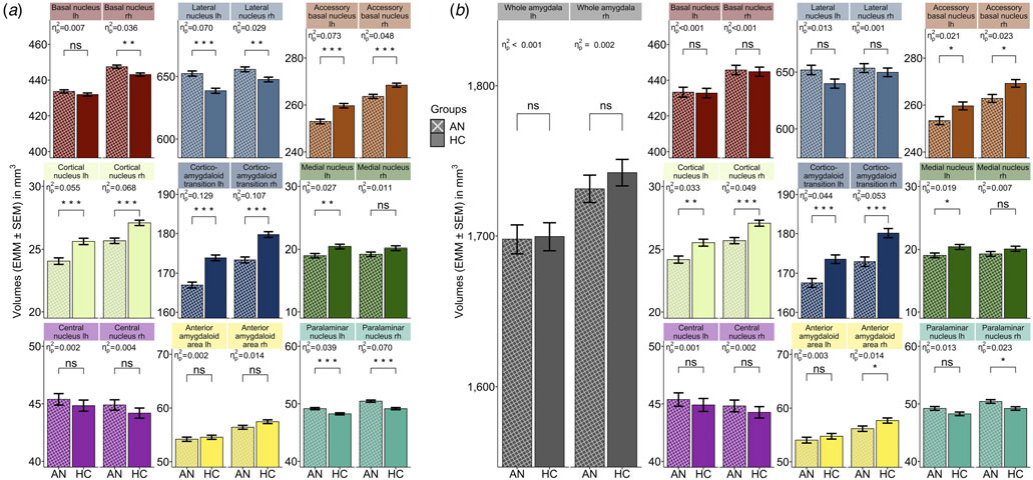
Bar graph visualization of GLM1 (a, whole amygdala volume-adjusted) and GLM2 (b, total subcortical GM volume-adjusted) for individual amygdala nuclei volumes in AN *v.* HC. AN, patients with acute anorexia nervosa; HC, healthy control participants; lh, left brain hemisphere; rh, right brain hemisphere; subcortical GM volume, total subcortical gray matter volume; GLM, general linear model. Bar graphs with error bars for study groups AN (*n* = 168) and age-matched HC (*n* = 168) displaying adjusted means (EMM, mm^3^) ± standard error of the mean (s.e.m.) of individual whole amygdala and amygdala nuclei volumes in separate brain hemispheres. Model estimates were obtained with either main GLM [performed separately for each amygdala (sub-)region, computed as *F* test: dfs = 1, 330]. (a) GLM1 covarying for age at date of research (linear and quadratic orthogonal polynomials), eTIV, and whole amygdala volume (lh, rh). (b) GLM2 covarying for age at date of research (linear and quadratic orthogonal polynomials), eTIV, and total subcortical GM volume [covariates in GLM1/2 (panels a/b) were grand mean-centered]. FDR-q, *p* values were multiple testing adjusted using false-discovery rate (Benjamini & Hochberg, 1995) across all amygdala nuclei and both GLM1 and GLM2 (whole amygdala adjusted separately using FDR but also across both GLMs). Significance levels for volume differences between study groups are stated as: ***q < 0.001; **q < 0.01; *q < 0.05; ns, non-significant. Effect size statistics are provided as partial *η*^2^ (Cohen, 1988).

Statistical analyses were conducted in R v4.1.1 (online SM 1.5) (R Core Team, 2022). Raw volumetric measures of amygdala (sub-)regions were approximately normally distributed in the study sample according to visual inspection and Shapiro–Wilk test (online SM 2.1, Fig. S2). All amygdala (sub-)region volumes were modeled using general linear models (GLMs) with study groups as the predictor, and a selection of covariates based on our research questions. To assess general/global AN-driven alterations in amygdala nuclei volumes, GLM0 (Fig. 1b) was adjusted for linear and quadratic orthogonal polynomials of participant age in line with recent ENIGMA studies due to evidence for nonlinear age effects on amygdala volumes (Chen et al., 2016; Han et al., 2020; Sämann et al., 2020; Vinke et al., 2018; Zugman et al., 2022). GLM0 was also adjusted for estimated total intracranial volume (eTIV), which is an established correction method of brain volumes for head size variation and recommended prior to any volumetric brain analysis (https://surfer.nmr.mgh.harvard.edu/fswiki/eTIV) (Malone et al., 2015; Sanfilipo, Benedict, Zivadinov, & Bakshi, 2004; Sargolzaei et al., 2015; Voevodskaya et al., 2014). In order to uncover specific effects of AN on amygdala nuclei against the background of AN-related (1) whole amygdala alterations, and (2) total subcortical GM alterations (King et al., 2018), we considered two relative GLMs: (1) GLM1 (Fig. 2a) included whole amygdala volume in addition to covariates from GLM0 to investigate subregional (i.e. within-amygdala) effects of AN; and (2) GLM2 (Fig. 2b) additionally covaried for total subcortical GM volume to examine amygdala nuclei alterations beyond AN-related generalized subcortical GM reductions. Multiple testing adjustment of *p* values using false-discovery rate (FDR) (Benjamini & Hochberg, 1995) was applied across all amygdala nuclei in GLM0, and across both relative GLMs (separately from GLM0). Supplementary GLMs (online SM 2.2) accounting for demographic/clinical variables that have been associated with amygdala alterations by previous research [GLM S1: SES, IQ, handedness, cigarette smoking (Durazzo, Meyerhoff, Yoder, & Murray, 2017; Elbejjani et al., 2019; Hao, Bertolero, & Farah, 2022; Szabo, Xiong, Lancaster, Rainey, & Fox, 2001; van der Plas, Boes, Wemmie, Tranel, & Nopoulos, 2010; Watkins, 2001)] and excluding AN participants (online GLM S2) with co-existing psychiatric diagnoses and/or psychoactive medication (Table 1) were implemented to confirm group differences from GLM0 (Frank, Favaro, Marsh, Ehrlich, & Lawson, 2018; King *et al*. 2018). We further investigated potential effects of AN subtype (restrictive/binge-purge), hydration status [urine-specific gravity (Biller et al., 2015; Streitbürger et al., 2012)], and oncotic pressure [serum albumin concentration (Wagner et al., 2006)] on amygdala nuclei volumes in AN (online SM 2.3).

To follow-up on amygdala nuclei significantly decreased in AN according to all GLM approaches, robust multiple linear regression analysis (RLM) (Huber, 1981) was performed within the AN group to test for associations between individual amygdala nuclei volumes and clinical measures, covarying for age, age^2^, and eTIV (online SM 2.4, Table S6). Clinical measures were grouped (online SM Table S6): (1) nutritional and neuroendocrine markers (BMI-SDS, log_10_-leptin), and (2) psychiatric severity markers [DOI, ED-specific symptoms (EDI-2 core and, exploratively, individual EDI-2 subscales ‘drive for thinness’, and ‘body dissatisfaction’), depressive symptoms (BDI-II total), anxiety (STAI(K)-trait), general psychiatric symptoms (SCL-90-R GSI)]. FDR-adjustment was applied across all RLMs per group of clinical measures. In HC, RLMs were estimated in an exploratory way with BMI-SDS and log_10_-leptin, exclusively [given pre-inclusion psychiatric screening of HC (i.e. low psychiatric symptom levels/variability, online SM 2.5, Table S7)].

## Results

### Sample characteristics

Study groups AN and HC did not differ in age, IQ, eTIV (Table 1), and handedness (online SM Table S2). Parental SES was higher in HC than AN (Table 1). Cigarette smoking prevalence was lower in AN than HC (online SM Table S2). As expected, AN had significantly lower BMI-SDS and, correspondingly, lower log_10_-leptin levels than HC, whereas ED-specific, depressive, trait anxiety, and general psychiatric symptom measures were markedly higher in AN (Table 1). Moreover, AN presented with reduced total subcortical GM volume [*t*(331) = 8.89, *p* < 0.001).

### Amygdala nuclei

When controlling for age and eTIV (GLM0, Fig. 1b), model estimates of whole amygdala (lh = −4.34%, rh = −4.22%) and most amygdala nuclei volumes were significantly smaller in AN than HC with maximum volumetric reductions in medial nucleus (lh = −10.70%, rh = −8.59%), cortical nucleus (lh = −9.33%, rh = −8.54%), corticoamygdaloid transition (lh = −7.98%, rh = −7.69%), and accessory basal nucleus (lh = −6.81%, rh = −6.00%; central and paralaminar nuclei rh were only nominally smaller in AN). Reported group differences from GLM0 remained robust (online SM Fig. S3B) after excluding AN with co-existing depressive, anxiety, obsessive-compulsive, and post-traumatic stress disorder diagnoses, and/or antidepressant pharmacotherapy (see Table 1). Likewise, findings were mostly unchanged when controlling for parental SES, IQ, handedness, and cigarette smoking in the main sample (except for the no longer significant volumetric reductions in lateral, central, and paralaminar nuclei lh, online SM Fig. S3A). Amygdala nuclei volumes were not related to AN subtype (online SM Table S3) and hydration status in AN (mostly within normal range, de-/hyperhydration in *n* = 2/6 AN, online SM Tables S4 and S5). Hypoalbuminemia did not occur in AN (online SM Table S4).

After adjusting for whole amygdala volume, significant within-amygdala differences between groups emerged for the following nuclei (GLM1, Fig. 2a): accessory basal and cortical nuclei lh and rh, corticoamygdaloid transition lh and rh, and medial nucleus lh were smaller in AN than HC. In contrast, basal nucleus rh, lateral and paralaminar nuclei lh and rh were larger in AN than HC relative to whole amygdala volume. The GLM2 approach, adjusting for total subcortical GM reductions in AN *v.* HC, yielded no group differences in whole amygdala volumes but smaller accessory basal and cortical nuclei lh and rh, corticoamygdaloid transition lh and rh, medial nucleus lh, and anterior amygdaloid area rh volumes in AN than HC. However, AN showed larger paralaminar nucleus rh than HC relative to total subcortical GM volume (Fig. 2b).

**Fig. 3.**
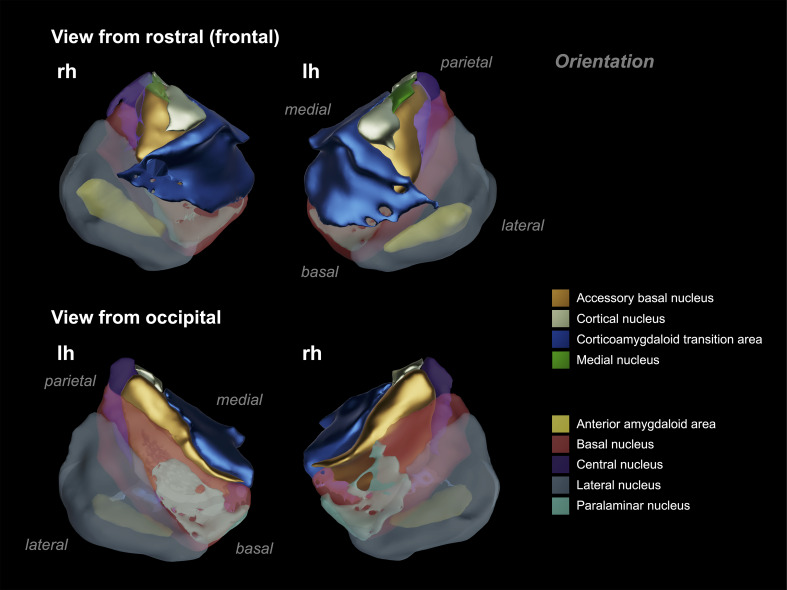
3D visualization of FreeSurfer-based amygdala subsegmentation into amygdala nuclei displaying group differences in AN *v.* HC. AN, patients with acute anorexia nervosa; HC, healthy control participants; lh, left brain hemisphere; rh, right brain hemisphere. Amygdala nuclei where significantly smaller volumes in AN *v.* HC were detected as overlapping findings according to GLM0 (eTIV-adjusted), GLM1 (whole amygdala volume-adjusted), and GLM2 (total subcortical GM volume-adjusted) are displayed with 100% opacity (namely, accessory basal nucleus lh and rh, cortical nucleus lh and rh, corticoamygdaloid transition lh and rh, and medial nucleus lh). Amygdala nuclei where group differences (i.e. smaller volumes in AN *v.* HC) were not significant at threshold FDR-q < 0.05 (according to at least one GLM approach) are displayed with 50% opacity. The figure shows a single-subject 3D model of amygdala nuclei (*n* = 9 lh, *n* = 9 rh), obtained from a preprocessed and normalized T1-weighted brain image of a typical patient with acute AN in the study sample, segmented via the FreeSurfer v7.1.1 automated amygdala subsegmentation tool, and rendered using the ‘Blender’ software (Blender Online Community, 2018). Orientation in the brain is given for amygdala lh (in gray/italics).

Notably, accessory basal, cortical, and medial nuclei, and corticoamygdaloid transition showed absolutely (GLM0) as well as relatively [i.e. compared to whole amygdala (GLM1) and total subcortical GM (GLM2) volume alterations] reduced volumes in AN *v.* HC, with almost perfect symmetry across brain hemispheres (except for medial nucleus: only lh affected). These subregions share an anatomically aggregated location in the rostral-medial amygdala (Fig. 3).

### Associations with clinical measures

**Fig. 4.**
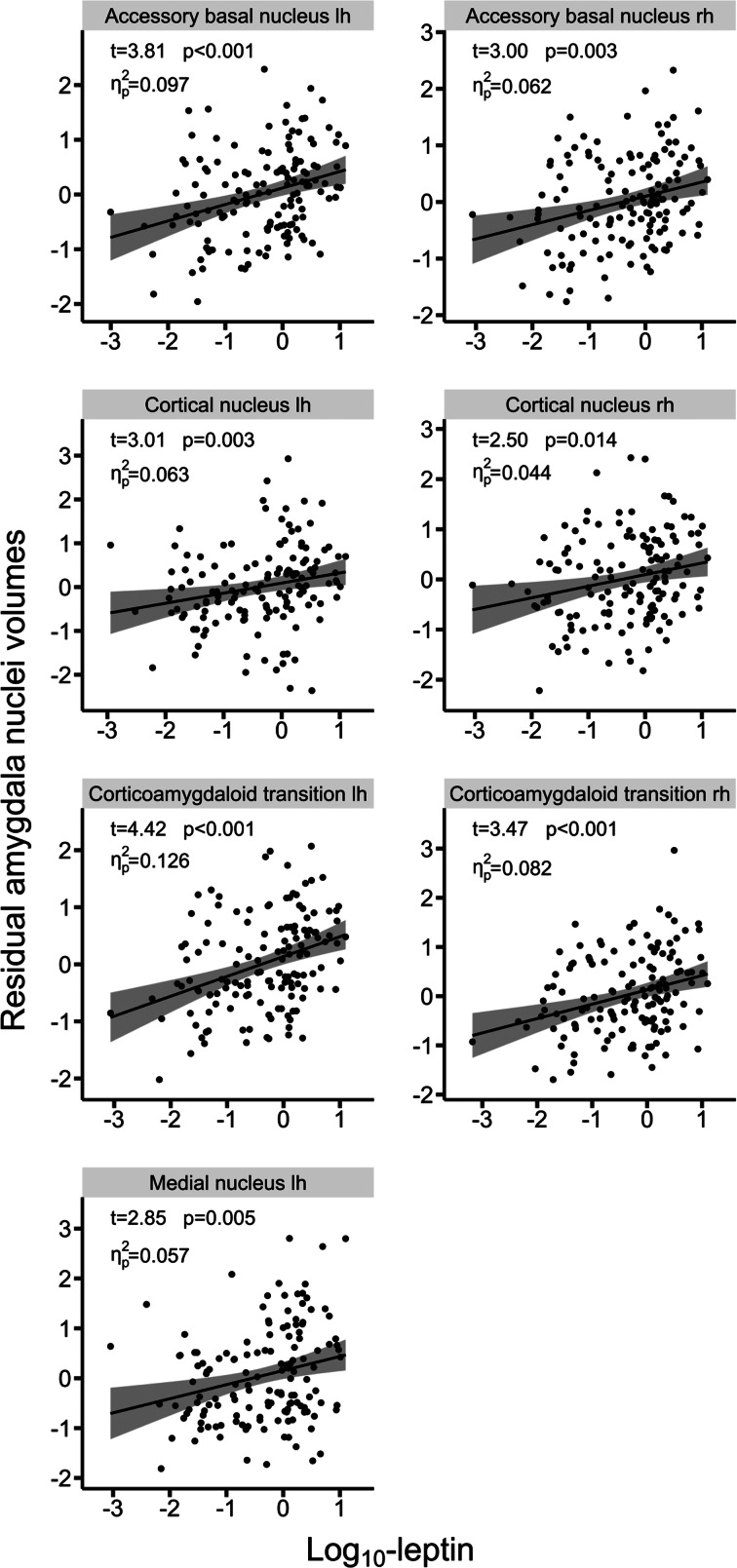
Scatter plots for associations between significantly reduced amygdala nuclei volumes and leptin concentrations in AN. Log_10_-leptin, logarithmically transformed (base 10) leptin concentration; lh, left brain hemisphere; rh, right brain hemisphere. Scatter plots with individual data points, linear regression lines, and 95% confidence intervals around the regression line (gray band) in the AN study group (plasma leptin measurement available in *n* = 142 of 168 AN) displaying associations between individual amygdala nuclei volumes that were significantly reduced in AN *v.* HC according to all GLM approaches (GLM0/1/2, Figs 1b–3) and log_10_-transformed plasma leptin concentrations [non-detectable leptin values <LOD of the leptin assay (*n* = 39 of 142) were multiple imputed using CLMI (Boss et al., 2019), associations were examined via RLMs applying M-estimation and Huber weighting for fitting via iterated re-weighted least squares]. Standardized residuals of amygdala nuclei volumes are plotted after adjustment of raw volume measures for age at date of research (linear and quadratic orthogonal polynomials) and eTIV using robust multiple linear regression. RLM statistics are provided as *t* value (unstandardized *β* divided by its standard error), unadjusted *p* value (computed via robust Wald *F* test), and effect size estimate partial *η*^2^ (Cohen, 1988) for each RLM for log_10_-leptin as the predictor [RLM formula: individual amygdala nuclei volumes (lh, rh) ~ log_10_-leptin + poly(age^1^, age^2^) + eTIV]. Log_10_-leptin remained a significant predictor of the plotted amygdala nuclei volumes after multiple testing FDR-adjustment, additional adjustment for BMI-SDS in follow-up RLMs, and after excluding all AN with leptin concentrations <LOD in a confirmatory analysis (online SM 2.4, Table S6).

RLMs in the AN study group examining all amygdala nuclei within the bilateral rostral-medial cluster [accessory basal, cortical, medial (lh) nuclei, and corticoamygdaloid transition, online SM Table S6] yielded significant positive associations of accessory basal nucleus lh and corticoamygdaloid transition lh volumes with BMI-SDS. Importantly, all rostral-medially clustered amygdala nuclei volumes, comprising accessory basal, cortical, medial nuclei, and corticoamygdaloid transition, were significantly and positively associated with log_10_-leptin levels in AN at medium strength (*η*_p_^2^ = 0.044–0.126, Fig. 4). To test for the unique effect of leptin above and beyond BMI-SDS effects in follow-up RLMs in AN, we covaried for BMI-SDS while orthogonalizing log_10_-leptin: after FDR-adjustment, log_10_-leptin explained additional variance in all amygdala nuclei volumes within the rostral-medial cluster (i.e. accessory basal, cortical, medial nuclei, and corticoamygdaloid transition). No significant associations between amygdala nuclei volumes and DOI or psychiatric symptom levels emerged in AN (online SM Table S6). Exploratory RLMs in HC did not reveal significant relationships between amygdala nuclei volumes and BMI-SDS or log_10_-leptin levels (online SM 2.5, Table S7).

## Discussion

In this first study to investigate amygdala substructure volumes in AN using sMRI, we found significant volumetric reductions of most amygdala nuclei in acutely underweight AN compared to HC. More importantly and going beyond previous findings of generally reduced whole amygdala volumes in AN (Burkert et al., 2019; Friederich et al., 2012; Giordano et al., 2001; Kappou et al., 2021; King et al., 2015; Zhang et al., 2018), a bilateral cluster located in the rostral-medial amygdala was particularly affected in AN as indicated by large effect sizes of group differences. This anatomical cluster comprised bilateral accessory basal and cortical nuclei, corticoamygdaloid transition, and left medial nucleus. Critically, the magnitudes of volumetric reductions in these subregions were more extensive relative to those observed for the whole amygdala and other subcortical GM volumes. In contrast, despite absolute volumetric reduction, basal nucleus rh as well as bilateral lateral and paralaminar nuclei were less affected in AN relative to whole amygdala and total subcortical GM volume reductions. These findings demonstrate differential alterations of individual nuclei within the amygdaloid complex suggestive of locally differing vulnerability to the effects of AN. Of note, lower leptin levels in patients with AN independently predicted greater volumetric reduction of amygdala nuclei within the rostral-medial cluster. This suggests an underlying or modulating role of hypoleptinemia, resulting from severe underweight, in relation to amygdala substructure alterations in AN, which might have relevant clinical implications.

Amygdala nuclei alterations observed in our study seem robust: they were largely independent of potential confounders including variation in demographic variables SES, IQ, handedness, and cigarette smoking (main results confirmed except for three nuclei outside of the rostral-medial cluster), AN subtype, co-existing psychiatric diagnoses, antidepressant use, and hydration status (Frank et al., 2018; King et al., 2018). Although generic amygdala substructure reductions may be expected informed by previous research on subcortical GM and whole amygdala alterations in AN (Bernardoni et al., 2016; Burkert et al., 2019; Eynde et al., 2012; Friederich et al., 2012; King et al., 2015; Monzon et al., 2017; Seitz et al., 2014, 2016; Su et al., 2021; Titova et al., 2013; Walton et al., 2022), degrees of absolute reduction of nuclei volumes within the amygdaloid complex appear rather heterogenous than uniform in AN as revealed by our study. In fact, they ranged from almost 11% (medial nucleus lh) to less than 2% (paralaminar nucleus rh) and showed focal maxima in subregions anatomically aggregated in the bilateral rostral-medial amygdala. Recent studies in major depressive disorder (Yao et al., 2020), obsessive-compulsive disorder (Zhang et al., 2020), post-traumatic stress disorder (Morey et al., 2020), and schizophrenia (Barth et al., 2021; Tesli et al., 2020; Zheng et al., 2019) have also documented differential alterations of amygdala subregions but spatial patterns differed from our findings. Importantly, despite limited comparability due to differing etiology, the medium-to-large effect sizes of amygdala nuclei reductions in acute AN found here substantially exceed the small effects previously reported under other neuropsychiatric conditions where widespread GM alterations are well-established, such as schizophrenia (van Erp et al., 2016). However, AN-related alterations in amygdala substructures do not reach the magnitude of chronic amygdala nuclei atrophy in neurodegenerative disorders such as Alzheimer's disease (20–30% tissue loss) (Cavedo et al., 2011) or frontotemporal dementia (35–50% volume loss) (Bocchetta, Iglesias, Cash, Warren, & Rohrer, 2019).

In addition to anatomically clustering in the rostral-medial amygdala, most of the nuclei, found to be decreased in AN not only in terms of absolute volume but also relative to reductions of whole amygdala and total subcortical GM volumes (Figs 2 and 3), have major histological and functional characteristics in common: they belong to the corticomedial amygdala subdivision (Fig. 1a) (LeDoux, 2007, 2008). Nuclei of this subdivision, namely cortical, medial nuclei, and corticoamygdaloid transition, are involved in olfactory information processing via their reciprocal connectivity with the olfactory cortex (Gutiérrez-Castellanos, Pardo-Bellver, Martínez-García, & Lanuza, 2014; LeDoux, 2007; Noto, Zhou, Yang, Lane, & Zelano, 2021; Oboti & Sokolowski, 2020). Accessory basal nucleus, also known as basomedial nucleus (LeDoux, 2007, 2008; McDonald, 2020; Watson, Paxinos, & Puelles, 2012), is anatomically and functionally closely related to the corticomedial amygdala subdivision (Gutiérrez-Castellanos et al., 2014; McDonald, 2020; Sah et al., 2003; Savander, Go, Ledoux, & Pitkänen, 1996) and plays critical roles in contextual/olfactory fear conditioning, aversion/reward processing (Cousens & Otto, 1998; Fanselow & LeDoux, 1999; LaBar & LeDoux, 1996; LeDoux, 2003; Yang et al., 2008), food motivation/palatability (Douglass et al., 2017; Haber, 2017; Kim et al., 2017; Lin, Mukherjee, Bernstein, & Katz, 2021; Petrovich, 2011; Simmons & Neill, 2009), and olfactory/visuospatial memory (Noto et al., 2021; Pratt & Mizumori, 1998; Riva, 2010; Yang & Wang, 2017). Accessory basal nucleus sends prominent projections to the ventral striatum, responsible for reward and emotional valence monitoring (Dieterich et al., 2021; Gutiérrez-Castellanos et al., 2014; Haber, 2017), as well as to the hippocampus and ento-/perirhinal cortex, serving as centers for memory formation (Pikkarainen & Pitkänen, 2001; Pikkarainen, Rönkkö, Savander, Insausti, & Pitkänen, 1999). Animal studies have further reported that medial nucleus regulates food intake-related and social behaviors via efferent projections to the hypothalamus with diverse vegetative, neuroendocrine, and homeostatic functions (Noto et al., 2021; Pardo-Bellver, Cádiz-Moretti, Novejarque, Martínez-García, & Lanuza, 2012; Sah et al., 2003). Based on the above-discussed functions of specific amygdala nuclei identified as severely affected in AN (Fig. 3) and accumulating evidence for brain structure–function relationships in neuropsychiatric disorders (Michael et al., 2011; Seidel et al., 2019), on the one hand, and clinical symptoms frequently occurring in patients with AN, on the other hand [e.g. body image distortion (Dakanalis et al., 2016; Favaro et al., 2012), specific fears like food (odor) aversion (Murray et al., 2018; Murray, Loeb, & Le Grange, 2016; Petrovich, 2011), altered olfactory sensitivity (Bentz et al., 2017; Mai et al., 2020)], we suspect that there might be associations between extensive volumetric reductions in rostral-medially clustered amygdala nuclei and AN-related symptoms. Trend-level correlations of whole amygdala volume with phobic anxiety and body image uncertainty were previously discovered in restrictive AN (Burkert et al., 2019). We measured selected psychiatric (ED-specific, depressive, anxious, and general) symptoms in the AN group retrospectively using self-report questionnaires and could not identify relationships with amygdala nuclei volumes of the rostral-medial cluster. In the context of differential amygdala nuclei alterations found here, it might be promising for future studies to evaluate further/other AN-related psychiatric symptoms [e.g. olfactory/disgust sensitivity (Glashouwer & de Jong, 2021)] and apply repeated real-time/-life assessments (Kwasnicka et al., 2021).

As anticipated based on evidence indicating that global GM reduction in acute AN rapidly returns to normal levels during short-term weight restoration (Bahnsen et al., 2022; King et al., 2018; Walton et al., 2022), we found that lower BMI-SDS in AN predicted smaller volumes of left hemispheric accessory basal nucleus and corticoamygdaloid transition (small effect size). Remarkably, the volumes of all rostral-medially clustered amygdala nuclei were more clearly associated with leptin levels than BMI-SDS in AN, hinting at specific leptin effects above and beyond the degree of underweight. In other words, leptin explained additional/other variance components of aforementioned amygdala nuclei volumes that were not attributable to BMI-SDS variation. This finding indicates predictive relevance of AN-related hypoleptinemia (Föcker et al., 2011; Hebebrand et al., 2007, 2022; Lawson & Klibanski, 2008) for the severity of rostral-medial amygdala nuclei reductions. Hence, we speculate that hypoleptinemia, in consequence of severe underweight, might be a possible (patho-)mechanism causally underlying and/or modulating the degree of amygdala substructure alterations in AN. This could, in a broader sense, offer novel insight into the neurobiology of striking and widespread, yet poorly understood, GM alterations in AN (King et al., 2018; Scharner & Stengel, 2019; Treasure et al., 2015; Zatorre et al., 2012). Basic research lends some support to our speculation by indicating leptin receptor expression in accessory basal and other amygdala nuclei and brain regions connected with baso-/corticomedial amygdala subdivisions (Fig. 1a) (Wada et al., 2014). Leptin signaling in the amygdaloid complex itself and innervating midbrain dopaminergic neurons interacts with mesolimbic reward pathways (Coccurello & Maccarrone, 2018; Fernandes et al., 2015), modulates anxiety-related behaviors (Liu, Perez, Zhang, Lodge, & Lu, 2011) and conditioned taste aversion (Han, Yan, Luo, Liu, & Wang, 2003) in rodents. Leptin also affects neural activity in the olfactory bulb (Sun et al., 2019) closely communicating with corticomedial amygdala nuclei (LeDoux, 2007; Sah et al., 2003). Furthermore, leptin promotes neurogenesis and synaptic plasticity in the amygdala-hippocampus formation in rodents (Bouret, 2010; Ge et al., 2018; Lu et al., 2006; Schepers et al., 2020). Translating to human research, leptin levels correlate with whole amygdala volume in female/male adults independent of BMI (Zonneveld et al., 2021) as well as with olfactory sensitivity (Fernandez-Garcia et al., 2017) and mood (Lawson et al., 2012) in females across the weight spectrum. According to case reports, leptin administration in leptin-deficient patients increased regional GM tissue concentration (Matochik et al., 2005) and, further, affected functional amygdala activation (Frank et al., 2013, 2011). In AN specifically, where the impact of hypoleptinemia on diverse neuroendocrine systems and AN-related symptoms is well-established (Ehrlich et al., 2009a, 2009b; Hebebrand et al., 2007, 2022; Lawson & Klibanski, 2008; Schneider et al., 2009), a greater leptin increase during weight restoration predicted less rumination about food at discharge independent of BMI (Fürtjes et al., 2018). Leptin might modulate food-related cognition in AN via its interactions with accessory basal and medial amygdala nuclei (Petrovich, 2011).

Clinical trials on recombinant human leptin administration in patients with AN have recently been encouraged (Hebebrand et al., 2022, 2019), given tolerability and potential beneficial effects of metreleptin on cognitive, emotional, and behavioral functions in AN as reported in case studies (Antel et al., 2021; Milos et al., 2020). Hyperactivity, mood, rumination about food, weight phobia, and even appetite/hunger seem to improve under metreleptin treatment applied in combination with inpatient nutritional rehabilitation (Antel et al., 2021; Milos et al., 2020). The associations between suppressed leptin levels and anatomically specific, severe GM reductions in the rostral-medial amygdala in AN may provide a novel mechanistic explanation for proposed metreleptin effects on core symptoms of AN considered to be linked to (dys-)functions of the severely affected amygdala nuclei unveiled by our study (Janak & Tye, 2015; LeDoux, 2007, 2008; Sah et al., 2003). However, given hypoleptinemia forms a protective adaptation to chronic starvation signaling the need to reduce energy expenditure and increase caloric intake (Hebebrand et al., 2007), possible negative effects of metreleptin treatment on body weight (Welt, Smith, & Mantzoros, 2004) may limit therapeutic use in AN and, therefore, need to be closely monitored in studies.

Less severe volumetric reductions (i.e. relative to whole amygdala and total subcortical GM volumes) of basal, lateral, central, and paralaminar amygdala nuclei might represent preserved or at least residual structural (and potentially functional) integrity. Human amygdala volume and neuron number normally increase by 40% and 11%, respectively, during adolescence (Avino et al., 2018) when AN episodes usually interfere. However, immature excitatory neurons in paralaminar nucleus migrating to basolateral nuclei well into adulthood have been revealed in post-mortem human amygdala specimens (Sorrells et al., 2019). Thus, our finding that paralaminar nucleus seems more resistant to the effects of AN might correspond to mostly unaffected neuronal plasticity within the amygdaloid complex in AN.

This is a sufficiently powered study in a large and homogeneous (regarding age, biological sex, ethnicity, IQ, eTIV) sample of AN and HC participants with sMRI acquisition at the same scanner following a standardized protocol throughout the entire study. However, our findings should be considered in the light of the following limitations: first, despite overall good test–retest reliability of FreeSurfer-based amygdala subsegmentation in within- and across-scanner comparisons (Kahhale, Buser, Madan, & Hanson, 2020; Morey et al., 2009; Quattrini et al., 2020), the applied FreeSurfer tool was designed using adult post-mortem and *in-vivo* brain samples (Saygin et al., 2017). Its performance is less well-established for adolescent brains (Schoemaker et al., 2016). We addressed this issue by developing a thorough QC procedure with extensive visual inspections and *a-priori* scan quality checks (Backhausen et al., 2016; Gilmore, Buser, & Hanson, 2021). Second, good numeric but lower spatial reliability has been proposed for single, smaller amygdala nuclei like medial and paralaminar nuclei (Kahhale et al., 2020; Quattrini et al., 2020). Thus, either cautious interpretation of corresponding FreeSurfer outputs, higher-resolution (>3 T) T1-weighted and additional T2-weighted scans, or more macro-level amygdala nuclei groupings (Oshri et al., 2019) have been suggested (Kahhale et al., 2020). Nonetheless, we analyzed amygdala nuclei individually and still recognized prominent bilateral groupings of severely reduced nuclei in AN bearing in mind that replication by future studies is needed to substantiate our results. Third, more sensitive measurement methods for leptin are available and could reduce left-censoring in AN [e.g. single-molecule array technology (Quanterix, 2016)]. We conducted CLMI, yielding efficient unbiased parameter estimates (Boss et al., 2019), to keep valuable data of AN with leptin concentrations <LOD of the leptin assay in our sample and preserve their natural variability. Finally, to clearly identify whether amygdala nuclei alterations form state-related reversible phenomena of AN or constitute trait markers, longitudinal investigations over the course of weight restoration and after long-term weight recovery are essential.

In conclusion, this study provides evidence for heterogeneous subregional alterations within the amygdaloid complex in acute AN showing local specificity in rostral-medially clustered amygdala nuclei of both brain hemispheres. Volumetric reductions in rostral-medial amygdala nuclei exceed other psychiatric disorders (e.g. schizophrenia) and even AN-related whole amygdala and total subcortical GM volume reductions. These anatomically clustered, severely affected nuclei are collectively involved in disorder-relevant functions (e.g. reward processing, food phobia, olfactory/visuospatial memory). Future studies will be important to determine whether the observed alterations contribute to neuropsychiatric symptom severity, treatment resistance, and long-term prognosis of AN (Fichter, Quadflieg, Crosby, & Koch, 2017). Our findings suggest that severe underweight and, in particular, associated hypoleptinemia in AN might have pathophysiological relevance for rostral-medial amygdala nuclei reductions. This adds evidence supporting the role of altered leptin signaling in AN distinctive of and going beyond simple measures of nutritional status, potentially modulating limbic system functions via hypoleptinemia-induced substructural changes within the amygdaloid complex.

## Supplementary material

For supplementary material accompanying this paper visit https://doi.org/10.1017/S0033291722003609. click here to view supplementary material
