# Supplementary material for: Differential alterations of amygdala nuclei volumes in acutely ill patients with anorexia nervosa and their associations with leptin levels
Source: Psychol Med. 2022 Dec 5;53(13):6288–303. doi: 10.1017/S0033291722003609 (PMC10358440; doi:10.1017/S0033291722003609)
Supplement: Supplementary file 1 [file S0033291722003609sup001.pdf]

Appendix: Supplementary Material to "Differential alterations of amygdala nuclei volumes in acutely ill patients with anorexia nervosa and their associations with leptin levels"

Authors

Marie-Louis Wronski, CandMed[a,b]

Daniel Geisler, MSc[a]

Fabio Bernardoni, PhD[a]

Maria Seidel, PhD[a]

Klaas Bahnsen, CandMed[a]

Arne Doose, MSc[a]

Jonas L. Steinhäuser, CandMed[a]

Franziska Gronow, MSc[a,c]

Luisa V. Böldt[a,d]

Franziska Plessow, PhD[b]

Elizabeth A. Lawson, MD[b]

Joseph A. King, PhD[a]

Veit Roessner, MD[e]

Stefan Ehrlich, MD, PhD[a,f]

[a] Translational Developmental Neuroscience Section, Division of Psychological and Social Medicine and Developmental Neurosciences, Faculty of Medicine, TU Dresden, Dresden, Germany.

[b] Neuroendocrine Unit, Department of Medicine, Massachusetts General Hospital and Harvard Medical School, Boston, MA, USA.

[c] Institute of Medical Psychology, Charité University Medicine Berlin, Berlin, Germany.

[d] Charité University Medicine Berlin, Berlin, Germany.

[e] Department of Child and Adolescent Psychiatry, Faculty of Medicine, University Hospital Carl Gustav Carus, TU Dresden, Dresden, Germany.

[f] Eating Disorder Treatment and Research Center, Department of Child and Adolescent Psychiatry, Faculty of Medicine, TU Dresden, Dresden, Germany.

Correspondence to Stefan Ehrlich, Division of Psychological and Social Medicine and Developmental Neurosciences, Faculty of Medicine, TU Dresden, Fetscherstraße 74, 01307 Dresden, Germany, Tel: +49 351 458-5214, Fax: +49 351 458-7206, E-mail: transden.lab@uniklinikum-dresden.de.

SM 1 Supplementary methods

SM 1.1 Participants

For all study groups (AN, patients with acute anorexia nervosa; HC, healthy control participants), additional exclusion criteria were applied – most importantly: a history of bulimia nervosa (BN) or binge-eating disorder; consumption of any psychoactive medications within four weeks prior to the study (except for selective serotonin reuptake inhibitors [SSRIs] and mirtazapine in the AN group: n=4 AN were under current treatment with SSRIs, n=1 AN was under treatment with mirtazapine); current substance abuse; any history of organic brain syndrome, schizophrenia, substance dependence, bipolar disorder, or psychosis not otherwise specified; current inflammatory, neurologic, or metabolic disorders; chronic illness that could affect appetite, eating behavior, or body weight. Further, study participants were excluded if they were pregnant or breast feeding, had anemia, or if their IQ was <85.

HC were excluded if they were currently underweight (current BMI <10th age percentile if younger than 18 years or <18.5kg/m^2 if 18 years and older) or obese (current BMI >94th age percentile if younger than 18 years or >28.0kg/m^2 if 18 years and older).

SM 1.2 Demographic and clinical measures

Parental (household) socioeconomic status (SES) (Patrick et al., 2004) is presented in Table 1 in the main article (range according to the German educational system: 0 [lowest], leaving school without graduation; 1, special-needs school graduation [“Sonderschulabschluss”]; 2, secondary school graduation [“Hauptschulabschluss”]; 3, intermediate secondary school graduation [“Realschulabschluss”]; 4, high school graduation [“Abitur”]; 5 [highest], university graduation [“Hochschulabschluss”]).

Regarding the educational level of study participants (i.e., not referring to their parents or guardians), 47 AN (27.98%) stated middle school as their highest educational degree, 117 AN (69.64%) high school, and 2 AN (1.19%) university (other degree in n=2 AN [1.19%]) according

to the German educational system. In HC, 17 participants (10.12%) stated middle school as their highest educational degree, 143 (85.12%) high school, and 6 (3.57%) university (other degree in n=2 HC [1.19%]). Regarding the current housing situation of study participants, 144 AN (85.71%) lived with their parents or guardians and 9 AN (5.36%) in their own household (housing situation not assessed in n=15 AN [8.93%]). In HC, 115 (68.45%) lived with their parents or guardians and 21 (12.50%) in their own household (housing situation not assessed in n=32 HC [19.05%]). Regarding current occupation, 140 AN (83.33%) were students, 1 AN (0.60%) was a volunteer worker, and 7 AN (4.17%) worked in non-academic professions (occupation not assessed in n=20 AN [11.90%]). In HC, 130 (77.38%) were students, 3 (1.79%) volunteer workers, and 3 (1.79%) worked in academic or non-academic professions (occupation not assessed in n=32 HC [19.05%]).

IQ of study participants was estimated with short versions of the German adaptation of the Wechsler Adult Intelligence Scale (WIE) (von Aster et al., 2006), or the Wechsler Intelligence Scale for Children (HAWIK-IV) (Petermann & Petermann, 2011) for study participants aged 15 years or younger. Handedness was assessed using six items of the Annett Scale of Hand Preference (Annett, 1970) which asks for handedness in daily life activities. Current cigarette smoking including quantitative cigarette consumption (number of cigarettes per day) was captured as part of our in-house semi-structured research interview.

SES, IQ, Annett Scale of Hand Preference total score, and current smoking status (smoker/non-smoker and number of cigarettes per day) were used as covariates in GLM S1 (SM 2.2, Table S2, Figure S3A).

The presence and severity of lifetime (from puberty) or current (last three months) eating-related psychopathology (including AN subtype and physical activity, see SM 1.5 and SM 2.3, Table S3) were evaluated in study participants using the expert form of the Structured Interview for Anorexia and Bulimia Nervosa (SIAB-EX) (Fichter & Quadflieg, 1999), a well-validated 87-item semi-standardized interview with good inter-rater reliability (mean κ=0.81 for current and κ=0.85 for past diagnosis) (Fichter & Quadflieg, 2001). SIAB-interviews were

conducted by clinically experienced and trained research assistants under the supervision of the attending child and adolescent psychiatrist. Co-existing psychiatric diagnoses other than eating disorders were derived according to standard practice from medical records (careful chart review including the consideration of medical and psychiatric history, physical examination, routine blood tests, urine analysis, and several psychiatric screening instruments) and confirmed by a board licensed child and adolescent psychiatrist with over ten years of clinical experience.

SM 1.3 Leptin measurements and multiple imputation procedure

To yield blood plasma for leptin measurements, fasting blood samples obtained from AN and HC were immediately processed as follows: addition of the serine protease inhibitor aprotinin, centrifugation (at ϑ=5°C and a=2500g for 15min), aliquotation into Eppendorf Tubes, storage at ϑ=-80°C until laboratory analysis.

Blood samples were processed in four batches at the same laboratory (Magdeburg/Germany 2014, 2015, 2017, and 2021, see SM 2.4 for exploratory analysis with dummy-coded batch covariates to account for [and exclude] potential batch effects). Plasma leptin concentrations were measured (as single measurement) using the same enzyme-linked immunosorbent assay (BioVendor Research and Diagnostic Products, Brno/Czech Republic) with an inter-assay variation coefficient of 5.6% and a lower limit of detection (LOD) of 0.20µg/L across all batches. For batches 2014, 2015, and 2017 (n=94 of 168 AN, n=90 of 168 HC assessed), however, leptin concentrations ≥0.05µg/L were measured according to the calibration curve and leptin concentrations ≥0.05µg/L to <0.20µg/L underwent subsequent statistical analyses as original/raw measures (i.e., were not imputed, occurred in n=6 of 94 AN), whereas leptin concentrations <0.05µg/L were imputed (occurred in n=19 of 94 AN). For batch 2021 (n=48 of 168 AN, n=66 of 168 HC assessed), leptin concentrations ≥0.20µg/L (=LOD) were measured (i.e., within detection range) and leptin concentrations <0.20µg/L were

imputed (occurred in n=20 of 48 AN). Plasma leptin concentrations below LOD did not occur in HC. Blood samples were not available in n=26 AN and n=12 HC.

Censored likelihood maximization based multiple imputation (CLMI) was applied to impute leptin values below LOD in AN (Boss et al., 2019). In total, 27.46% of leptin measurements in AN were left-censored non-detects. Due to the differing minimum leptin concentrations measured across leptin batches (0.05µg/L for batches before 2021, 0.20µg/L for batch 2021), our CLMI model accounted for both thresholds (0.05µg/L and 0.20µg/L) to guarantee that imputed leptin values fell below batch-specific thresholds. First, CLMI constructed a conditional censored likelihood distribution function of logarithmically transformed leptin values ($\log_{10}$-leptin), given the outcome variable (amygdala nuclei volumes which were significantly reduced in AN vs. HC according to all GLM approaches [GLM 0/1/2], Figures 1–3 in the main article) and a matrix of covariates. Omitting the outcome variable in the imputation model is not recommended since it would result in a bias toward the null when a true association between $\log_{10}$-leptin and amygdala nuclei volumes adjusted for covariates exists (Boss et al., 2019; van Buuren & Groothuis-Oudshoorn, 2011). Second, n=50 random samples were iteratively drawn from the fitted truncated distribution of $\log_{10}$-leptin within the limits 0 < random sample < LOD of the leptin assay (limits are stated on the original scale of leptin concentrations, n=50 samples per examined amygdala nucleus, i.e., n=350 imputed datasets in total). Third, the analysis model (see SM 1.5 and SM 2.4, robust linear models with $\log_{10}$-leptin as the predictor) was fitted separately on the n imputed datasets (CLMI-imputed $\log_{10}$-leptin in AN was also used in two-samples t-test for group differences in $\log_{10}$-leptin between AN and HC, see SM 1.5 and Table 1 in the main article). Note that our imputation and analysis models were consistent, i.e., the imputation model included at least the same (and additional, see below) variables with identical transformations as the analysis model ($\log_{10}$-transformation of leptin, z-transformation of the other variables in both models to prevent multicollinearity and achieve numeric stability of multiple imputation due to largely differing original scales of magnitude). Our imputation model was more general than the analysis model by including auxiliary predictors that were either significantly associated (correlated) with $\log_{10}$-

leptin or relevant based on previous research (Boss et al., 2019). Fourth, pooling of the n imputed datasets for statistical inference was applied according to Rubin's (1987) combination rules (based on univariate pooled Wald test following a t- or F-distribution to infer p-value, degrees of freedom were calculated dependent on the number of imputations, the between-imputation variance, and the within-imputation variance) (Rubin, 1987; van Buuren & Groothuis-Oudshoorn, 2011).

The following CLMI imputation model was used in our study:

- **CLMI formula:** log_{10}-leptin ~ individual amygdala nuclei volumes (lh, rh) + $^{\perp}$poly(age^1, age^2) + eTIV + BMI-SDS + BMI_{min} + DOI + EDI-2 core score + BDI-II total score + STAI(K) trait anxiety score + AN subtype (SIAB-EX) + physical activity (SIAB-EX);
- **Amygdala nuclei volumes** (outcome variable, significantly reduced nuclei in AN vs. HC according to GLM 0/1/2 in the main article were analyzed individually resulting in n=7*50=350 imputed datasets): Accessory basal nucleus lh and rh, cortical nucleus lh and rh, corticoamygdaloid transition lh and rh, medial nucleus lh;
- **Predictors** in CLMI imputation and analysis models: age, $^{\perp}age^2$, eTIV, and BMI-SDS;
- **Auxiliary predictors and reason for inclusion in CLMI imputation model:**
    - ***Minimal lifetime BMI (BMI_{min}):*** significantly correlated with log_{10}-leptin in AN,
    - ***Duration of illness (DOI):*** theoretical background and previous research (more severe endocrine alterations in AN with longer DOI (Hebebrand et al., 2007; Schorr & Miller, 2017)),
    - ***EDI-2 core score:*** core eating disorder symptoms were significantly correlated with log_{10}-leptin in AN,
    - ***BDI-II total score:*** depressive symptoms were significantly correlated with log_{10}-leptin in AN,
    - ***STAI(K) trait anxiety score:*** trait anxiety symptoms were significantly correlated with log_{10}-leptin in AN,

- ***AN subtype*** (SIAB-EX: restrictive, binge-purge): theoretical background and previous research (evidence for differing leptin levels across AN subtypes (Eddy et al., 2015)),
- ***Physical activity*** (SIAB-EX: five ordered levels from no to very frequent excessive physical activity): theoretical background and previous research (lower leptin levels are linked to higher physical activity in AN (Ehrlich et al., 2009; Holtkamp et al., 2006)).

SM 1.4 Quality control (QC) procedure

FreeSurfer's automated cortical reconstruction stream and whole brain segmentation ("recon-all") (Fischl, 2004; Fischl et al., 2002) was applied to all T1-weighted input MRI scans including, among others, the following processes: registration, motion correction, realignment, skull stripping, Talairach transformation, intensity normalization, tessellation, averaging and analysis. Subsequently, subjects were analyzed with the combined amygdala and hippocampus subsegmentation tool (Saygin et al., 2017) available in FreeSurfer version 7.1.1 using its cross-sectional processing stream (https://surfer.nmr.mgh.harvard.edu/fswiki/HippocampalSubfieldsAndNucleiOfAmygdala).

Given there is still no standardized QC algorithm for FreeSurfer-based amygdala subsegmentation, the QC procedure used by the current study was developed in accordance with and as an extension to ENIGMA (Enhancing Neuro Imaging Genetics through Meta Analysis) QC recommendations for hippocampal subfields (Sämann et al., 2020). Since amygdala and hippocampus are segmented with one combined FreeSurfer tool (see above), we correspondingly performed a combined QC procedure for both amygdala and hippocampus subsegmentations. Our multi-stage QC procedure comprised HTML-snapshot-based visual checks of amygdala (and hippocampus) subsegmentations of all subjects, followed by an outlier-guided dynamic/detailed visual inspection of suspicious subjects according to the study group-wise detection of statistical outliers by means of combination criteria (volume and bilateral symmetry outliers). QC was conducted by two trained raters

(Marie-Louis Wronski, Luisa V. Böldt) with ratings “1” for “inclusion” vs. “9” for “exclusion” of the amygdala (and hippocampus) (sub-)segmentation of a certain subject. See Figure S1 for a flow scheme summarizing our QC stages. Actual QC performance was preceded by a visual QC test phase (via FreeSurfer’s viewing application FreeView) on randomly selected, unsuspicious subjects (n=20) from patient and control groups to become familiar with the appearance of successfully applied amygdala (and hippocampus) subsegmentations and the natural variance across subsegmentations. After the initial QC test phase, each rater independently inspected further n=20 randomly selected subjects from patient and control groups (mixture of unsuspicious and suspicious subjects without and with statistical outliers). Independent ratings were then compared to determine interrater reliability via Cohen’s κ (Cohen, 1960): κ=0.76, which lies within the range classified as substantial agreement between raters (Landis & Koch, 1977).

Subjects eligible for amygdala (and hippocampus) QC were required to (A) have good overall scan quality of their T1-weighted MRI scans assessed via signal-to-noise (SNR) and contrast-to-noise ratio (CNR) measures from FreeSurfer’s recon-all output statistics (outlier subjects with >2.698*SD (Tukey, 1977) downward deviation from the group mean of SNR or CNR were a-priori excluded due to low scan quality), and (B) have passed our internal general cortical and subcortical QC procedure (e.g., no severe skull inclusion or misapplied general subcortical segmentation), adapted from ENIGMA guidelines and consisting of a visual HTML-snapshot QC and, eventually, FreeView-based inspection in case of peculiarities.

After a-priori exclusions due to scan quality or general QC rating and prior to any outlier diagnostics, all subjects eligible for amygdala (and hippocampus) QC were visually checked for misapplied subsegmentation using HTML-snapshots in line with current ENIGMA recommendations (Figure S1) (Sämann et al., 2020). At this stage, the raters paid particular attention to the following aspects: (A) binary hippocampal mask should be visible, (B) hippocampal fissure should lie within the hippocampal mask, and (C) no severe incompleteness of the colored amygdala and hippocampus subsegmentation overlays. In case of observed peculiarities, the referring subsegmentations were re-inspected dynamically

via FreeView. Segmentation failure led to exclusion of the whole amygdala (and hippocampus) (sub-)segmentation of a subject.

For the additional dynamic and more detailed visual inspection of suspicious (i.e., outlier) subjects via FreeView, combination criteria for statistical outliers in amygdala (and hippocampus) subsegmentations were introduced (each amygdala and hippocampal [sub-]region was regarded separately, Figure S1): (A) subjects with one or more “extreme” volume or symmetry outlier(s) (symmetry outlier diagnostics via a lateralization index as applied by previous ENIGMA studies (Kong et al., 2020)) deviating >4.721*SD from the group mean would undergo FreeView-QC (equals 1.quartile - 3*interquartile range or 3.quartile + 3*interquartile range in a boxplot (Tukey, 1977)), and (B) subjects with two or more “mild” volume or symmetry outliers deviating >2.698*SD from the group mean would also undergo FreeView-QC (equals 1.quartile - 1.5*interquartile range or 3.quartile + 1.5*interquartile range in a boxplot (Tukey, 1977)). The latter cutoff of *“two or more mild outliers”* was set empirically based on the distribution of our data (ca. 15% of all subjects in the initial sample [$n_{initial}$=688 scans] met this criterion), feasibility, and the fact that isolated outlier observations are unusual in case of segmentation failure (Sämann et al., 2020). The cutoff was verified with a mathematical approach (binomial distribution, see below *).

Regarding our detailed visual QC performance via FreeView, the hippocampal fissure was defined as the starting point for visual QC. Multiple slices of the desired amygdala (and hippocampus) subsegmentation were inspected dynamically, simultaneously for left and right brain hemispheres and in different planes (axial, coronal, sagittal) with the help of FreeView zoom options and down- or upregulation of the opacity of the colored segmentation overlay (so that structures in the intensity-corrected T1-scan became visible). The following observations were considered as relevant for the decision whether to include or exclude a suspicious subject: (A) color allocation to subregions of the amygdala and hippocampus according to the color legend in FreeView and missing or incomplete parts of the color overlay, (B) large and unambiguous artifacts affecting the whole amygdala (and hippocampus) segmentation (e.g., obliterations, blurred margins due to motion, severe white matter

inclusions), (C) severe violations against bilateral segmentation symmetry, and (D) artifacts affecting single subregions (e.g., spared regions [“holes”] due to tissue misclassification or cystic formations, merging or extending subregions forming “islands” in neighboring subregions). QC ratings (1, inclusion vs. 9, exclusion) for each subject including screenshots of and comments on potential peculiarities were documented by both raters (Marie-Louis Wronski, Luisa V. Böldt) and subjects with unclear segmentation success were subsequently discussed in an expert QC meeting. In case of critical artifacts or segmentation failure, the whole amygdala (and hippocampus) (sub-)segmentation of a certain subject was excluded (i.e., not single subregions).

Table S1 summarizes exclusions or, respectively, inclusions of subjects at each stage of our QC procedure and, further, shows that the number of exclusions at each QC stage did not differ between study groups (AN, HC).

* Binomial distribution approach: The probability $P(X \geq x)$ of “≥cutoff” outlier observations per subject was set to approximately 5%. The probability of a single outlier observation was p=0.007 (for thresholds: mean ± 2.698*SD according to the Gaussian Normal Distribution). The number of observations or tests per subject was n=96 (10 amygdala [sub-]regions per hemisphere, 10 amygdala symmetry tests; 22 hippocampal [sub-]regions per hemisphere, 22 hippocampus symmetry tests). The cutoff was computed as $X \geq 2$ volume or symmetry outlier observations per subject via $X \sim B(n, p)$.

Flow scheme illustrating our quality control (QC) procedure for amygdala (and hippocampus) subsegmentations

0. General specifications

(A) Image quality outliers (SNR, signal-to-noise ratio; CNR, contrast-to-noise ratio) were a-priori excluded. Threshold: mean - 2.698*SD.

(B) Subject had to pass our general cortical and subcortical QC (snapshot- and FreeView-based visual QC, in line with ENIGMA standards).

(C) We applied a combined QC procedure for amygdala and hippocampus subsegmentations.

(D) We performed an outlier-guided visual QC (FreeView) of suspicious subjects, preceded by HTML-snapshot inspections of amygdala (and hippocampus) sub-segmentations of all subjects (after a-priori exclusions). Study group-wise outlier detection was based on combination criteria (volume and bilateral symmetry).

1. Visual QC test phase via FreeView

Amygdala (and hippocampus) subsegmentations of a small list (n=20) of randomly selected subjects from different study groups (without outliers) were visually inspected (dynamic FreeView-based inspection including all planes) as a reference for successful segmentation and for acceptable variance across unsuspicious segmentations.

2. HTML-snapshot-based visual QC of all amygdala (and hippocampus) subsegmentations

Rapid snapshot QC for global impression of successful or misapplied segmentation. QC focus: Binary hippocampal mask visible? Hippocampal fissure within the hippocampal mask?

⇒ In case of peculiarities, segmentation was inspected extensively and in all planes via FreeView. Very suspicious subjects (segmentation failure) were excluded.

3. Selection of subjects with statistical volume or symmetry outliers in amygdala (and hippocampus) subsegmentations for additional/follow-up visual QC via FreeView

Statistical outlier criteria (study group-wise detection): volume outliers (amygdala and hippocampal [sub-]regions and brain hemispheres separately) and bilateral symmetry outliers (amygdala and hippocampal [sub-]regions separately, based on lateralization index LI=(lh-rh)/(lh+rh)).

(A) Subjects with ≥1 extreme volume or symmetry outlier(s) underwent dynamic FreeView-QC. Thresholds: mean ± 4.721*SD.

(B) Subjects with ≥2 mild volume or symmetry outliers underwent dynamic FreeView-QC. Thresholds: mean ± 2.698*SD.

4. Visual QC via FreeView of selected amygdala (and hippocampus) subsegmentations

Dynamic visual QC of amygdala (and hippocampus) subsegmentations for outlier subjects using FreeView and including the inspection in all planes (axial, coronal, and sagittal).

⇒ Suspicious segmentations were discussed in an expert QC meeting. Decision regarding the inclusion or exclusion of suspicious subjects.

Figure S1. QC flow scheme. *Abbreviations: QC, quality control; SD, standard deviation; LI, lateralization or laterality index; lh, left brain hemisphere; rh, right brain hemisphere.* Flow scheme illustrating the combined amygdala and hippocampus subsegmentation (FreeSurfer v7.1.1) QC procedure that was applied by the current study. The shell script used to prepare dynamic visual inspection contained commands to open the following images in FreeSurfer's viewing application FreeView: nu.mgz (intensity normalized volume/raw image), lh.hippoAmygLabels-T1.v21.mgz and rh.hippoAmygLabels-T1.v21.mgz (standard subsegmentations of left and right hemispheric hippocampal subfields and amygdala nuclei at subvoxel resolution of 0.333mm). The scripts (shell, MATLAB scripts) for HTML-snapshot QC of amygdala and hippocampus subsegmentations were kindly provided by the ENIGMA (Enhancing Neuro Imaging Genetics through Meta Analysis) Major Depressive Disorder working group (project: hippocampal subfields, Philipp Sämann).

Table S1. Summary table of QC results.

		n_x / n_{total}	%	**Chi-squared test statistics** (df=1): test statistic; p-value
**A-priori exclusions**				
n(subjects) a-priori removed due to **scan quality outliers** (SNR and/or CNR, threshold: mean - 2.698*SD)	Sample	8 / 688	1.16	
	AN	3 / 237	1.27	0.87; 1.000[a]
	HC	5 / 451	1.11	
n(subjects) a-priori removed due to artifacts in **general cortical and subcortical QC**	Sample	23 / 680	3.38	
	AN	10 / 234	4.27	0.50; 0.479
	HC	13 / 446	2.91	
**Case selection for visual QC** (… following HTML-snapshot-based QC of all subjects after a-priori exclusions, n=657) → dynamic FreeView-based QC for scans with statistical outliers according to volume and symmetry outlier criteria[b]				
n(subjects) selected for dynamic visual QC (via FreeView)	Sample	102 / 657	15.53	n/a
**Visual QC exclusions** (HTML and/or FreeView)				
n(subjects) excluded during visual QC	Sample	22 / 657	3.35	
	AN	5 / 224	2.23	0.84; 0.360
	HC	17 / 433	3.93	
**Included subjects after visual QC (post-QC sample)**				
n(subjects) that have passed all QC stages[c]	Sample	635 / 688	92.30	
	AN	219 / 237	92.41	n/a
	HC	416 / 451	92.24	

Abbreviations: QC, quality control; AN, patients with acute anorexia nervosa; HC, healthy control participants; SNR, signal-to-noise ratio; CNR, contrast-to-noise ratio; SD, standard deviation; n/a, not assessed. Summarized results (exclusions and final inclusions) of our combined amygdala and hippocampus subsegmentation (FreeSurfer v7.1.1) QC procedure. Number of excluded or included subjects (i.e., MRI scans) at each QC stage (n_x) and total number of available subjects (i.e., MRI scans) (n_{total}) are given for the complete sample and study group-wise (AN, HC). The absolute counts of exclusions at each QC stage did not differ between study groups according to Chi-squared test or [a]Fisher's exact test (for frequencies/counts <5). [b]Visual QC comprised two stages: (A) HTML-snapshot QC of amygdala (and hippocampus) subsegmentations for all subjects after a-priori exclusions, (B) additional/follow-up dynamic FreeView-QC of amygdala (and hippocampus) subsegmentations for outlier subjects after study group-wise detection of volume outliers (separately for individual amygdala and hippocampal [sub-]regions and brain hemispheres) and symmetry outliers (separately for individual amygdala and hippocampal [sub-]regions via lateralization index for left [lh] and right [rh] hemispheric volumes: LI=(lh - rh)/(lh + rh) (Kong et al., 2020)) according to the following cutoffs and thresholds: subjects with ≥1 extreme ("far out") volume or symmetry outlier(s) (thresholds: mean ± 4.721*SD (Tukey, 1977)) and subjects with ≥2 mild volume or symmetry outliers (thresholds: mean ± 2.698*SD (Tukey, 1977)) were selected for FreeView-based QC. [c]Note that the post-QC sample (n=219

AN, n=416 HC) differs from the main (final) study sample for statistical analyses (n=168 AN, n=168 HC). The post-QC sample still included multiple (>1) QC-approved scans of individual AN and HC participants because they often participated in several research studies. If that was the case, only one QC-approved scan per AN and HC participant was chosen based on a predefined hierarchy: (A) date of research, i.e., first AN episode and scan closest to the initiation of nutritional rehabilitation (all scans were acquired within 96h after admission) were preferred in AN to avoid confounding by early effects of inpatient treatment, (B) amygdala and general cortical/subcortical segmentation quality, i.e., best QC rating out of the QC-approved scans per participant was preferred in HC. Subsequently, a subset of HC participants was selected through age-matching to patients with AN to optimize group comparisons (using an optimal pair matching algorithm (Hansen & Klopfer, 2006)).

SM 1.5 Flow scheme illustrating main and supplementary analyses

1. Preliminaries
- Multi-stage QC of amygdala/hippocampus subsegmentations (FS v7.1.1)
- AN and HC often participated in several research studies → selection of n=1 QC-approved scan per participant based on first AN episode and first date of research (in AN) and best QC rating (in HC) → sample including only n=1 scan/participant: n=168 AN, n=286 HC
- Pairwise age-matching of HC to AN → **n=168 AN, n=168 HC**

Initial study sample

688 MRI scans/subjects
237 AN study group
451 HC study group

Exclusions (-)

- QC: **-** 18 AN, **-** 35 HC
- Selection of n=1 scan/ participant: **-** 51 AN, **-** 130 HC
- Age-matching: **-** 118 HC (unmatched)

Main study sample (age-matched)

336 study participants
168 AN study group
168 HC study group

2. Data distribution and sample characteristics
- Visualization of raw amygdala (nuclei) volumes from lh and rh via histograms
- Descriptive statistics: demographic variables (incl. IQ, handedness, smoking) and clinical measures (incl. BMI-SDS, leptin, psychiatric symptoms); AN subtype, DOI, psychiatric comorbidities/psychoactive medication

3. Censored likelihood maximization based multiple imputation of plasma leptin concentrations below LOD in AN
- **CLMI imputation model:** Log_{10}-leptin ~ individual amygdala nuclei volumes (lh, rh) + $^{\perp}$poly(age^1, age^2) + eTIV + BMI-SDS + BMI_{min} + DOI + EDI-2 core score + BDI-II total score + STAI(K) trait anxiety score + AN subtype + physical activity
- **Steps: 1)** CLMI using imputation model (n=50 iterations), **2)** Analysis model (t-test [see 4.]), RLMs [see 6.]) applied to each imputed dataset, **3)** Pooling of estimates from all models in 2) for overall statistical inference

4. Two-samples t-tests for group differences (AN vs. HC) in relevant demographic variables and clinical measures

5. General linear model (GLM) approaches for group differences (AN vs. HC) in whole amygdala and individual amygdala nuclei volumes in separate brain hemispheres (lh, rh)
- Computed as analysis of covariance/F-test; Multiple testing adjustment: FDR
- *Sub-analysis in AN: GLMs to examine potentially confounding effects of AN subtype and altered hydration status*

GLM0 (absolute)

→ *Leading question: Are there general/global effects of AN on individual amygdala nuclei (absolute approach, adjusted only for age and head size)?*

Whole amygdala volume (lh, rh) or individual amygdala nuclei volumes (lh, rh) ~ study groups + $^{\perp}$poly(age^1, age^2) + eTIV

Supplementary GLMs

→ *Leading question: Are findings from GLM0 reproducible when accounting for potential confounders (SES, IQ, handedness, smoking), and when excluding AN with psychiatric comorbidities and/or SSRI or mirtazapine intake?*

GLM S1
Whole amygdala volume (lh, rh) or individual amygdala nuclei volumes (lh, rh) ~ study groups + $^{\perp}$poly(age^1, age^2) + eTIV + SES + IQ + hand preference + cigarette smoking + n(cigarettes/day)

GLM S2
Whole amygdala volume (lh, rh) or individual amygdala nuclei volumes (lh, rh) ~ post-exclusion groups + $^{\perp}$poly(age^1, age^2) + eTIV

Main GLMs

GLM1 (relative)
→ *Leading question: Are there within-amygdala effects of AN on individual amygdala nuclei (i.e., relative to variation in whole amygdala volume)?*

Individual amygdala nuclei volumes (lh, rh) ~ study groups + $^{\perp}$poly(age^1, age^2) + eTIV + whole amygdala volume (lh, rh)

GLM2 (relative)
→ *Leading question: Are there specific effects of AN on amygdala nuclei volumes beyond general/global subcortical gray matter reduction in AN (i.e., relative to variation in total subcortical GM volume)?*

Whole amygdala volume (lh, rh) or individual amygdala nuclei volumes (lh, rh) ~ study groups + $^{\perp}$poly(age^1, age^2) + eTIV + total subcortical GM volume

6. Multiple regression analysis: associations between amygdala nuclei volumes and clinical measures in patients with AN
- Clinical measures (2 groups): **nutritional and neuroendocrine markers –** BMI-SDS and leptin (log_{10}-leptin); **psychiatric severity markers –** DOI, EDI-2 core symptoms, BDI-II total, STAI(K) trait anxiety, SCL-90-R GSI
- **Robust linear models (RLMs)** in AN: Individual amygdala nuclei volumes (lh, rh) ~ clinical measure + $^{\perp}$poly(age^1, age^2) + eTIV
  → *RLMs with predictor **log_{10}-leptin**: follow-up BMI-SDS adjustment, confirmatory analysis for leptin ≥LOD, batch effects*
- Multiple testing adjustment: FDR across all amygdala nuclei within one group of clinical measures

Abbreviations: QC, quality control; FS, FreeSurfer; AN, patients with acute anorexia nervosa; HC, healthy control participants; lh, left brain hemisphere; rh, right brain hemisphere; SES, socioeconomic status; IQ, intelligence quotient; BMI-SDS, body mass index standard deviation score; DOI, duration of illness; LOD, lower limit of detection of the leptin assay (0.20µg/L); CLMI, censored likelihood multiple imputation; log_{10}-leptin, logarithmically transformed (base 10) leptin concentration; $^{\perp}$poly(age^1, age^2), linear and quadratic orthogonal polynomials of age at date of research due to evidence for nonlinear age effects on amygdala volumes (Chen et al., 2016; Han et al., 2020; Kurth et al., 2019; Pomponio et al., 2020; Sämann et al., 2020; Vinke et al., 2018), orthogonalization using the "poly()" function in the R base package "stats v4.1.1" (part of R v4.1.1) (R Core Team, 2022) to prevent multicollinearity (Chen et al., 2016); eTIV, estimated total intracranial volume; BMI_{min}, minimum lifetime BMI; EDI-2 core, averaged score comprising the core subscales "drive for thinness", "body dissatisfaction", and "bulimia" of Eating Disorder Inventory-2; BDI-II, Beck Depression Inventory-II; STAI, State-Trait Anxiety Inventory (for participants aged ≥15 years); STAIK, State-Trait Anxiety Inventory for Children (for participants aged <15 years); SCL-90-R GSI, Global Severity Index of the Symptom Checklist-90-Revised; FDR, false discovery rate; SSRI, selective serotonin reuptake inhibitor; subcortical GM volume, total subcortical gray matter volume.

GLMs/RLMs are displayed in formula style (Wilkinson & Rogers, 1973): dependent variable (volume of whole amygdala or individual amygdala nuclei, separately for lh and rh) ~ independent variable/predictor (GLMs: study groups, RLMs: clinical measure) + covariates (further independent variables, note that grand mean centering by subtracting the mean value of the full study sample from the value of an individual participant was applied to continuous independent variables in GLMs including AN and HC, whereas mean centering by subtracting the mean value of the AN or HC study group from the value of an individual AN or HC participant was applied to continuous independent variables in GLMs/RLMs including only AN or HC). RLMs were performed with M-type estimators and Huber weighting (psi-function) for fitting by iterated re-weighted least squares (IWLS) considered as robust against outliers and non-normal error distribution (Venables & Ripley, 2002). The choice of this resistant regression method for our analyses was rather conservative since significant deviations from multiple linear regression assumptions did not occur in our data according to visual inspection of residual plots. For CLMI of log_{10}-leptin values below LOD in AN, continuous independent variables in the imputation model were z-transformed via subtracting the mean value of the AN study group from the value of an individual AN participant and dividing the resulting difference by the standard deviation of the AN study group to improve numeric stability given differing original scales of the variables in the CLMI model. AN subtype was included as a binary variable with two unordered levels (restrictive, binge-purge) and physical activity as an ordinal variable with five ordered levels (from no to very frequent excessive physical activity) in the CLMI model.

Statistical analyses were conducted using R v4.1.1 (R Core Team, 2022) with packages "MatchIt" (Ho et al., 2011) for age-matching, "lodi" (Boss & Rix, 2020) and "mice" (van Buuren & Groothuis-Oudshoorn, 2011) for CLMI-based multiple imputation, "car" (Fox & Weisberg, 2019) for GLM computation, "MASS" (Venables & Ripley, 2002) for RLM computation, and "ggplot2" (Wickham, 2016) for visualization.

SM 2 Supplementary results

SM 2.1 Distribution of raw amygdala (nuclei) volumes

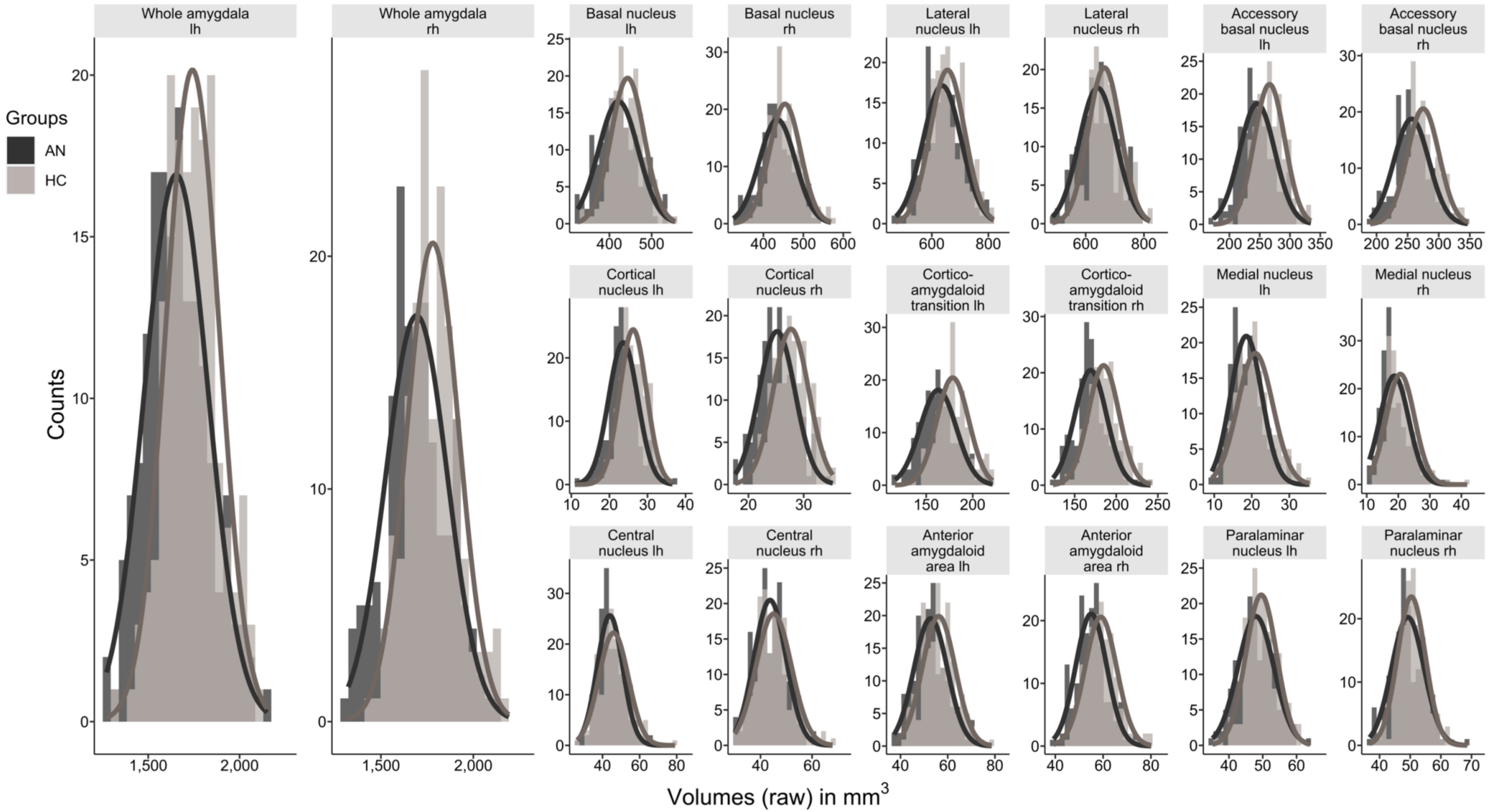

Figure S2. Histograms with normal curves visualizing the distribution of raw amygdala (nuclei) volumes in the study sample. *Abbreviations: AN, patients with acute anorexia nervosa; HC, healthy control participants; lh, left brain hemisphere; rh, right brain hemisphere.* Histograms with normal curves for study groups AN (n=168) and age-matched HC (n=168) displaying the distribution (counts) of raw volumes (mm^3) of whole amygdala and individual amygdala nuclei in separate brain hemispheres. Visual inspection revealed that raw volumes of whole amygdala and individual amygdala nuclei were approximately normally distributed. Exploratively, histograms were supplemented by QQ plots (not shown), Shapiro-Wilk tests (for normality), and Levene tests (for equality of variances).

SM 2.2 Supplementary GLMs

Supplementary GLMs were performed to verify findings from GLM0 in the main article (Figure 1B) while accounting for further potential demographic and clinical confounders that have been associated with structural and/or functional amygdala alterations.

GLM S1 (Figure S3A)

GLM S1 was applied in the main study sample (n=168 AN, n=168 HC) and adjusted for the following covariates (see Table S2 for descriptive statistics):

- Covariates from GLM0 (in the main article, Figure 1B): age, $^{\perp}age^{2}$, and eTIV;
- **Socioeconomic status** (SES, parental [household] SES in our study) reported to be positively correlated with amygdala and hippocampus volumes in adolescents and young adults (Hao et al., 2022; Merz et al., 2018; Noble et al., 2012): included as an ordinal covariate in GLM S1 (range 0–5);
- **Intelligence quotient** (IQ) shown to be positively correlated with total brain volume (Nave et al., 2019; Pietschnig et al., 2015), amygdala (van der Plas et al., 2010) and hippocampus (Amat et al., 2008) volumes: included as a continuous covariate in GLM S1;
- **Handedness** due to evidence for differing ratios of left and right hemispheric amygdala and hippocampus volumes between handedness categories (right, mixed, left) (Szabo et al., 2001; Watkins, 2001) and for age-related amygdala and hippocampus atrophy depending in its progression on the strength of handedness (Cherbuin et al., 2011): included as a continuous covariate in GLM S1 (Annett Scale of Hand Preference total score (Annett, 1970) in line with literature (Annett, 1994; Cherbuin et al., 2011));
- **Cigarette smoking** reported to be associated with smaller gray matter volume (Elbejjani et al., 2019) and accelerated age-related volume loss (Durazzo et al., 2017) in the amygdala: two covariates were included in GLM S1 to represent current

smoking (in the last six months before study participation) due to the high proportion of non-smokers in the study sample (Table S2), i.e., high prevalence of structural zeros (He et al., 2017):

- Binary indicator of current smoking status (0=smoker vs. 1=non-smoker),
- Continuous covariate for number of cigarettes per day (including zeros for non-smokers) so that the GLM equation simplifies to the effect of n(cigarettes)/day for smokers (indicator=0, count variable≠0), and to the effect of binary smoking status for non-smokers (indicator=1, count variable=0).

GLM S2 (Figure S3B)

GLM S2 was applied after the exclusion of AN (n=24) with co-existing psychiatric diagnoses and/or psychoactive medication in the last six months before study participation (see Table 1 in the main article). HC from the main sample were selected (n=144) to match the post-exclusion AN group (n=144) for age (using the same optimal pair matching algorithm as in the main article/main sample (Hansen & Klopfer, 2006)). Covariates from GLM0 (in the main article, Figure 1B) were also included in GLM S2: age, $^{\perp}age^2$, and eTIV.

Based on previous research, the following co-existing psychiatric diagnoses and psychoactive medication in AN were considered as relevant and excluded in GLM S2:

- **Major depressive disorder** known to be associated with volumetric reductions of the amygdala and hippocampus formation (Dusi et al., 2015; Hamilton et al., 2008; Stratmann et al., 2014);
- **Anxiety or panic disorder** with evidence for smaller amygdala volumes (Hayano et al., 2009) and frequently observed amygdala hyperactivation in functional MRI (Etkin & Wager, 2007);
- **Obsessive-compulsive disorder** given some evidence for volumetric reduction of the amygdala (Pujol et al., 2004);

- **Post-traumatic stress disorder** shown to co-exist with amygdala and hippocampus volume alterations (mostly reduction of whole amygdala volume, whereas amygdala nuclei were described to be differentially altered) (Logue et al., 2018; Morey et al., 2020);
- **Antidepressants** (only SSRIs and mirtazapine were included in this study) reported to affect amygdala volume (potentially by promoting neurogenesis accompanied by volumetric increase (Dusi et al., 2015; Hamilton et al., 2008) but direction of change is yet unclear (Szeszko et al., 2004)) and amygdala activation in task-based functional MRI (Young et al., 2020).

Table S2. Supplementary demographic variables.

	**n**	**Sample**<br>Mean ± SD<br>*Median ± IQR*		**Analyses**		**Handedness:**<br>Chi-squared test	**Smoking:**<br>Fisher's exact test
	**AN / HC**	**AN**	**HC**	**U**	**p**	**p**	**p**
**Handedness**							
Annett Scale of Hand Preference: total score	164 / 163	1.04 ± 2.64<br>*0.00 ± 1.00*	1.23 ± 2.84<br>*0.00 ± 1.00*	14355.50	0.156	n/a	n/a
Right-side preference	143 / 140						
Mixed handedness	11 / 12	n/a	n/a	n/a	n/a	0.942	n/a
Left-side preference	10 / 11						
**Current cigarette smoking** (last six months before study participation)							
Smoker	3 / 19	n/a	n/a	n/a	n/a	n/a	0.001
Non-smoker	164 / 149						
n(cigarettes)/day	167 / 168	0.01 ± 0.08	0.20 ± 1.26	n/a	n/a	n/a	n/a
n(cigarettes)/day in the smoker group	3 / 19	0.51 ± 0.42<br>*0.57 ± 0.42*	1.73 ± 3.46<br>*0.43 ± 1.29*	31.50	0.811	n/a	n/a

Abbreviations: AN, patients with acute anorexia nervosa; HC, healthy control participants. Number of participants and, where applicable, mean value ± standard deviation (SD) and *median ± interquartile range (IQR)* for each variable and study group (AN, HC) are shown. Group differences in hand preference total score and n(cigarettes)/day in the smoker group were tested using nonparametric Mann-Whitney U-tests due to unequal sample sizes and deviations from normality. As test statistics, U-value and p-value (normal approximation with continuity correction) are stated. Group differences in counts of handedness and smoking status were tested using Chi-squared or Fisher's exact tests (the latter for frequencies/counts <5), and p-value is stated.

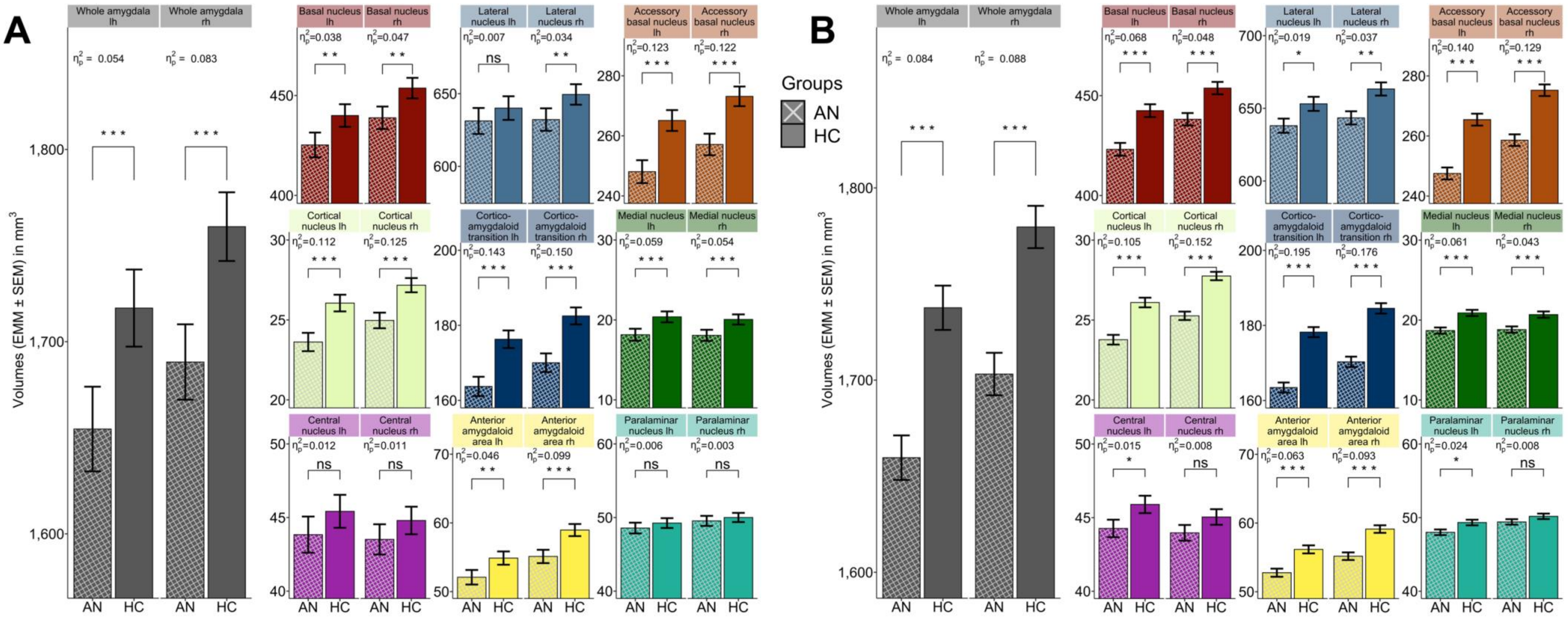

Figure S3. Bar graph visualization of GLM S1 (A, adjusted for demographic and clinical covariates) and GLM S2 (B, after exclusion of AN with co-existing psychiatric diagnoses and/or antidepressant medication) for individual amygdala (nuclei) volumes in AN vs. HC. *Abbreviations: AN, patients with acute anorexia nervosa; HC, healthy control participants; lh, left brain hemisphere; rh, right brain hemisphere; GLM, general linear model.* Bar graphs with error bars for study groups AN (**A**, n=123 of 168 main sample AN with available covariate data; **B**, n=144 AN post-exclusion) and age-matched HC (**A**, n=117 of 168 main sample HC with available covariate data; **B**, n=144 HC age-matched to post-exclusion AN) displaying adjusted means (EMM, mm^3) ± standard error of the mean (SEM) of individual whole amygdala and amygdala nuclei volumes in separate brain hemispheres. Model estimates were obtained with either supplementary GLM (performed separately for each amygdala [sub-]region, computed as F-test: **A**,

df=1,230; **B**, df=1,283): **A – GLM S1** covarying for age at date of research (linear and quadratic orthogonal polynomials), eTIV, parental (household) socioeconomic status (Patrick et al., 2004), IQ (note that socioeconomic status and IQ were correlated at Spearman’s rho=0.24, df=238, p<0.001 [in all participants in GLM S1, i.e., across study groups]; both socioeconomic status and IQ were treated as nuisance covariates in GLM S1 and, thus, included simultaneously), Annett Scale of Hand Preference total score (Annett, 1970), and current cigarette smoking (modeled as one binary covariate to indicate smoking status [smoker, non-smoker] and one continuous covariate for n(cigarettes)/day; continuous covariates were grand mean-centered except for n(cigarettes)/day due to structural zeros); **B – GLM S2** covarying for age at date of research (linear and quadratic orthogonal polynomials) and eTIV in the post-exclusion sample (covariates were grand mean-centered). FDR-q: p-values were multiple testing adjusted using false discovery rate (FDR) (Benjamini & Hochberg, 1995) across all amygdala nuclei but separately for each supplementary GLM (whole amygdala adjusted separately using FDR). Significance levels for volume differences between study groups are stated as: ***, q<0.001; **, q<0.01; *, q<0.05; ns, nonsignificant. Effect size statistics are provided as partial η^2 (Cohen, 1988).

SM 2.3 AN subtype, hydration status, and serum albumin concentration in AN

Supplementary GLM analysis in the AN study group (Table S3) revealed that there was no significant effect of AN subtype (n=142 restrictive AN, n=22 binge-purge AN, subtype not assessed in n=4 AN) on amygdala (nuclei) volumes.

Table S3. GLM for amygdala (nuclei) volumes predicted by AN subtype in the AN study group.

Amygdala (sub-)region	F	df	FDR-q	η_p^2
**Whole amygdala**				
Whole amygdala lh	2.38	1,159	0.125	0.015
Whole amygdala rh	3.17	1,159	0.125	0.020
**Amygdala nuclei**				
Accessory basal nucleus lh	5.41	1,159	0.191	0.033
Accessory basal nucleus rh	2.60	1,159	0.346	0.016
Anterior amygdaloid area lh	0.01	1,159	0.963	<0.001
Anterior amygdaloid area rh	1.33	1,159	0.376	0.008
Basal nucleus lh	1.96	1,159	0.365	0.012
Basal nucleus rh	2.50	1,159	0.346	0.015
Central nucleus lh	1.05	1,159	0.425	0.007
Central nucleus rh	1.47	1,159	0.371	0.009
Cortical nucleus lh	2.26	1,159	0.346	0.014
Cortical nucleus rh	0.01	1,159	0.963	<0.001
Corticoamygdaloid transition lh	6.24	1,159	0.191	0.038
Corticoamygdaloid transition rh	4.62	1,159	0.198	0.028
Lateral nucleus lh	0.65	1,159	0.505	0.004
Lateral nucleus rh	2.84	1,159	0.346	0.018
Medial nucleus lh	1.79	1,159	0.365	0.011
Medial nucleus rh	0.00	1,159	0.963	<0.001
Paralaminar nucleus lh	0.75	1,159	0.501	0.005
Paralaminar nucleus rh	1.54	1,159	0.371	0.010

Abbreviations: AN, patients with acute anorexia nervosa; lh, left brain hemisphere; rh, right brain hemisphere; GLM, general linear model. GLM for individual whole amygdala and amygdala nuclei volumes (in alphabetical order) in separate brain hemispheres predicted by AN subtype (factor with two levels: restrictive vs. binge-purge) in the AN study group and covarying for age at date of research (linear and quadratic orthogonal polynomials) and eTIV (n=164 of 168 AN with available data, GLM performed separately for each amygdala [sub-]region, computed as F-test: df=1,159; covariates were mean-centered). As model statistics, F-value, degrees of freedom (df), FDR-q, and effect size estimate partial η^2 (Cohen, 1988) are stated. FDR-q: p-values were multiple testing adjusted using false

discovery rate (FDR) (Benjamini & Hochberg, 1995) across all amygdala nuclei (whole amygdala adjusted separately using FDR).

Nutritional assessment of patients with AN (circa one hour before MRI scanning) included clinical measurements of urine specific gravity from first-morning specimens to assess hydration status (Baron et al., 2015) and of serum albumin concentration from fasting blood samples. Albumin regulates oncotic (colloidal osmotic) pressure responsible for intra-/ extravascular fluid shifts (Wagner et al., 2006). Both de- or hyperhydration and hypoalbuminemia have been shown to affect gray matter volume (Biller et al., 2015; Streitbürger et al., 2012; Wagner et al., 2006).

Regarding hydration status, dehydration occurred in n=2 AN, and hyperhydration in n=6 AN (see Table S4 for reference range). Hydration status was not significantly associated with amygdala (nuclei) volumes in the AN study group (Table S5).

Regarding serum albumin concentration, hypoalbuminemia did not occur in the AN study group (see Table S4 for reference range).

Table S4. Urine specific gravity and serum albumin concentration in the AN study group.

**Parameter**	n	Mean ± SD	Range (min–max)	Reference range (literature)
Urine specific gravity	128	1.010 ± 0.003	1.002–1.022	1.006–1.020 (Popowski et al., 2001)
Serum albumin concentration (g/L)	135	50.44 ± 3.89	40.10–63.00	≥35 (lower limit) (Jassam et al., 2020)

Number of participants with available data (n), mean value ± standard deviation (SD), and range (minimum–maximum) are stated for each clinical variable in the AN study group (n_{total}=168). Reference/normal range according to literature is given.

Table S5. GLM for amygdala (nuclei) volumes predicted by urine specific gravity in the AN study group.

Amygdala (sub-)region	F	df	FDR-q	η_p^2
**Whole amygdala**				
Whole amygdala lh	0.16	1,123	0.879	0.001
Whole amygdala rh	0.02	1,123	0.879	<0.001
**Amygdala nuclei**				
Accessory basal nucleus lh	0.17	1,123	0.974	0.001
Accessory basal nucleus rh	0.20	1,123	0.974	0.002
Anterior amygdaloid area lh	1.05	1,123	0.974	0.008
Anterior amygdaloid area rh	0.14	1,123	0.974	0.001
Basal nucleus lh	0.18	1,123	0.974	0.001
Basal nucleus rh	0.13	1,123	0.974	0.001
Central nucleus lh	1.21	1,123	0.974	0.010
Central nucleus rh	0.01	1,123	0.974	<0.001
Cortical nucleus lh	0.05	1,123	0.974	<0.001
Cortical nucleus rh	0.00	1,123	0.974	<0.001
Corticoamygdaloid transition lh	0.67	1,123	0.974	0.005
Corticoamygdaloid transition rh	0.67	1,123	0.974	0.005
Lateral nucleus lh	0.66	1,123	0.974	0.005
Lateral nucleus rh	0.19	1,123	0.974	0.002
Medial nucleus lh	0.01	1,123	0.974	<0.001
Medial nucleus rh	0.82	1,123	0.974	0.007
Paralaminar nucleus lh	0.56	1,123	0.974	0.005
Paralaminar nucleus rh	0.00	1,123	0.974	<0.001

Abbreviations: AN, patients with acute anorexia nervosa; lh, left brain hemisphere; rh, right brain hemisphere; GLM, general linear model. GLM for individual whole amygdala and amygdala nuclei volumes (in alphabetical order) in separate brain hemispheres predicted by urine specific gravity (hydration status) in the AN study group and covarying for age at date of research (linear and quadratic orthogonal polynomials) and eTIV (n=128 of 168 AN with available urine samples, GLM performed separately for each amygdala [sub-]region, computed as F-test: df=1,123; continuous independent variables were mean-centered). As model statistics, F-value, degrees of freedom (df), FDR-q, and effect size estimate partial η^2 (Cohen, 1988) are stated. FDR-q: p-values were multiple testing adjusted using false discovery rate (FDR) (Benjamini & Hochberg, 1995) across all amygdala nuclei (whole amygdala adjusted separately using FDR).

SM 2.4 Robust linear regression analysis for associations between amygdala nuclei volumes and clinical measures in AN

Table S6. RLMs in the AN study group.

RLMs in AN (formula): Individual amygdala nuclei volumes (lh, rh) ~ clinical measure + ⊥poly(age[1], age[2]) + eTIV

**Amygdala nuclei volumes** (significantly reduced in AN according to all assessed comparisons/models)		**Clinical measures**								
		**(A) Nutritional and neuroendocrine markers**				**(B) Psychiatric severity markers**				
		**BMI-SDS** (n=168)	**Log_{10}-leptin** (n=142)[a]	*Follow-up: **log_{10}-leptin**, adjusted for BMI-SDS (n=142)* [b]	*Confirmatory analysis for leptin ≥LOD: **log_{10}-leptin** (n=97)* [c]	**DOI** (n=164)	**EDI-2 core** (n=164)[d]	**BDI-II total** (n=166)	**STAI(K) trait anxiety** (n=141)	**SCL-90-R GSI** (n=165)
Accessory basal nucleus lh	β	4.61	8.90	*5.75*	*20.00*	-0.13	0.02	0.00	-0.12	-3.03
	95% CI	[1.49, 7.72]	[4.27, 13.54]	*[0.40, 11.10]*	*[9.45, 30.55]*	[-0.35, 0.10]	[-0.53, 0.57]	[-0.33, 0.34]	[-0.47, 0.22]	[-9.33, 3.26]
	t	2.90**	3.81**	*2.13**	*3.71***	-1.08	0.07	0.02	-0.68	-0.95
	p	0.004	<0.001	*0.035*	*<0.001*	0.274	0.943	0.988	0.493	0.345
	η^2_p	0.049	0.097	*0.033*	*0.132*	0.008	<0.001	<0.001	0.003	0.006
Accessory basal nucleus rh	β	2.21	6.55	*5.63*	*11.94*	-0.09	-0.33	-0.16	-0.11	-2.39
	95% CI	[-0.74, 5.16]	[2.15, 10.94]	*[0.47, 10.79]*	*[2.49, 21.38]*	[-0.31, 0.12]	[-0.84, 0.17]	[-0.46, 0.15]	[-0.42, 0.21]	[-7.92, 3.15]
	t	1.47	3.00**	*2.16**	*2.48**	-0.88	-1.29	-0.99	-0.67	-0.85
	p	0.144	0.003	*0.033*	*0.015*	0.375	0.198	0.322	0.503	0.398
	η^2_p	0.013	0.062	*0.034*	*0.062*	0.005	0.010	0.006	0.003	0.004
Cortical nucleus lh	β	0.40	1.00	*0.76*	*2.31*	0.00	0.01	0.00	-0.01	-0.47
	95% CI	[-0.05, 0.85]	[0.37, 1.63]	*[0.02, 1.50]*	*[0.68, 3.95]*	[-0.03, 0.03]	[-0.06, 0.09]	[-0.04, 0.05]	[-0.06, 0.04]	[-1.30, 0.36]
	t	1.74	3.01**	*2.02**	*2.77**	-0.09	0.38	0.08	-0.44	-1.11
	p	0.080	0.003	*0.045*	*0.006*	0.927	0.709	0.941	0.660	0.265
	η^2_p	0.019	0.063	*0.030*	*0.080*	<0.001	<0.001	<0.001	0.001	0.008
Cortical nucleus rh	β	0.16	0.73	*0.74*	*1.37*	-0.01	-0.06	-0.01	0.01	-0.36
	95% CI	[-0.23, 0.55]	[0.16, 1.29]	*[0.08, 1.40]*	*[0.02, 2.72]*	[-0.04, 0.02]	[-0.13, 0.00]	[-0.05, 0.03]	[-0.03, 0.05]	[-1.09, 0.37]
	t	0.81	2.50*	*2.21**	*1.98**	-0.58	-1.87	-0.36	0.46	-0.96
	p	0.416	0.014	*0.029*	*0.049*	0.556	0.062	0.721	0.647	0.336

	η_p^2	0.004	0.044	*0.035*	*0.042*	0.002	0.022	<0.001	0.002	0.006
Corticoamygdaloid transition lh	β	3.33	7.06	*5.29*	*12.83*	-0.09	0.11	0.03	-0.06	-1.56
	95% CI	[1.15, 5.52]	[3.90, 10.22]	*[1.55, 9.03]*	*[5.51, 20.15]*	[-0.24, 0.07]	[-0.27, 0.49]	[-0.21, 0.27]	[-0.29, 0.17]	[-5.80, 2.67]
	t	2.99**	4.42***	*2.80**	*3.43***	-1.09	0.58	0.23	-0.52	-0.72
	p	0.003	<0.001	*0.006*	*<0.001*	0.274	0.563	0.820	0.600	0.470
	η_p^2	0.052	0.126	*0.055*	*0.114*	0.008	0.002	<0.001	0.002	0.003
Corticoamygdaloid transition rh	β	1.91	5.48	*4.84*	*10.89*	-0.15	-0.10	-0.06	-0.06	-1.44
	95% CI	[-0.19, 4.01]	[2.35, 8.61]	*[1.17, 8.51]*	*[3.60, 18.18]*	[-0.30, 0.00]	[-0.47, 0.27]	[-0.29, 0.16]	[-0.29, 0.16]	[-5.46, 2.57]
	t	1.78	3.47**	*2.61**	*2.93**	-1.95	-0.54	-0.54	-0.54	-0.70
	p	0.076	<0.001	*0.010*	*0.004*	0.050	0.594	0.593	0.592	0.481
	η_p^2	0.019	0.082	*0.048*	*0.085*	0.024	0.002	0.002	0.002	0.003
Medial nucleus lh	β	0.36	1.12	*0.92*	*2.26*	0.00	0.02	0.04	0.00	-0.09
	95% CI	[-0.18, 0.90]	[0.37, 1.87]	*[0.03, 1.80]*	*[0.29, 4.23]*	[-0.04, 0.04]	[-0.07, 0.12]	[-0.02, 0.09]	[-0.05, 0.06]	[-1.14, 0.96]
	t	1.30	2.85**	*2.05**	*2.25**	-0.18	0.48	1.20	0.15	-0.17
	p	0.192	0.005	*0.043*	*0.027*	0.857	0.633	0.231	0.882	0.865
	η_p^2	0.010	0.057	*0.030*	*0.052*	<0.001	0.001	0.009	<0.001	<0.001

Abbreviations: RLM, robust linear model; AN, patients with acute anorexia nervosa; lh, left brain hemisphere; rh, right brain hemisphere; $^{\perp}$poly(age^1, age^2), linear and quadratic orthogonal polynomials of age at date of research due to evidence for nonlinear age effects on amygdala volumes (Chen et al., 2016; Han et al., 2020; Kurth et al., 2019; Pomponio et al., 2020; Sämann et al., 2020; Vinke et al., 2018), orthogonalization to prevent multicollinearity (Chen et al., 2016); eTIV, estimated total intracranial volume; BMI-SDS, body mass index standard deviation score; log_{10}-leptin, logarithmically transformed (base 10) leptin concentration; DOI, duration of illness; EDI-2 core, averaged score comprising the core subscales "drive for thinness", "body dissatisfaction", and "bulimia" of Eating Disorder Inventory-2; BDI-II, Beck Depression Inventory-II; STAI, State-Trait Anxiety Inventory (for participants aged ≥15 years); STAIK, State-Trait Anxiety Inventory for Children (for participants aged <15 years); SCL-90-R GSI, Global Severity Index of the Symptom Checklist-90-Revised.

RLMs in the AN study group (n_{total}=168, available n given in each column) for individual amygdala nuclei volumes (in alphabetical order) that were significantly reduced in AN vs. HC according to all GLM approaches (GLM0/1/2, Figures 1–3 in the main article), predicted by selected clinical measures (grouped: [A] – nutritional and neuroendocrine markers, [B] – psychiatric severity markers) and covarying for age at date of research (linear and quadratic orthogonal polynomials) and eTIV (each table cell represents an individual RLM, continuous independent variables were mean-centered using the mean of the AN study group). Censored likelihood multiple imputation (CLMI) (Boss et al., 2019) was applied for left-censored leptin values in AN below the lower limit of detection (LOD=0.20µg/L) of the leptin assay (see SM 1.3 for details on the imputation procedure). For log_{10}-leptin as the predictor in RLMs, continuous variables were z-transformed using the mean and standard deviation of the AN study group according to the CLMI imputation model. As model statistics, unstandardized regression coefficient of the predictor (β) and 95% confidence interval (CI), t-value (equals unstandardized β divided by its standard error), unadjusted p-value (p computed via robust Wald F-test), and effect size estimate partial η^2 (Cohen, 1988) of the predictor are stated for each RLM. FDR-q: p-values were multiple testing adjusted using false discovery rate (FDR) (Benjamini & Hochberg, 1995) across all RLMs per group of clinical measures. Significant FDR-adjusted regression findings are flagged as: ***, q<0.001; **, q<0.01; *, q<0.05.

[a]**Exploratory RLMs controlling for leptin batch effects** (RLM formula: individual amygdala nuclei volumes [lh, rh] ~ log_{10}-leptin + $^{\perp}$poly[age^1, age^2] + eTIV + leptin batch, all four batches 2014, 2015, 2017, and 2021 were included as dummy-coded variables, n=142 AN): After FDR-adjustment, log_{10}-leptin remained a significant independent predictor of all analyzed amygdala nuclei volumes (Accessory basal nucleus lh: t=3.79, p_{raw}<0.001; Accessory basal nucleus rh: t=3.18, p_{raw}=0.002; Cortical nucleus lh: t=3.22, p_{raw}=0.002; Cortical nucleus rh: t=2.63, p_{raw}=0.010; Corticoamygdaloid transition lh: t=4.45, p_{raw}<0.001; Corticoamygdaloid transition rh: t=3.50, p_{raw}<0.001; Medial nucleus lh: t=2.63, p_{raw}=0.010), and batch covariates were nonsignificant in all RLMs. Thus, potentially confounding effects of leptin batches could be excluded.

[b]**Follow-up BMI-SDS-adjusted RLMs**: The unique effect of leptin, adjusted for BMI-SDS, was investigated for amygdala nuclei where nominally significant associations with log_{10}-leptin were detected (follow-up RLM formula: individual amygdala nuclei volumes [lh, rh] ~ BMI-SDS + $^{\perp}log_{10}$-leptin + $^{\perp}$poly[age^1, age^2] + eTIV; log_{10}-leptin was orthogonalized to BMI-SDS via the Gram-Schmidt process (Leon et al., 2013) to prevent collinearity with BMI-SDS and examine the effect of leptin on amygdala nuclei volumes above and beyond the effect of BMI-SDS, i.e., the variance component of log_{10}-leptin that is orthogonal to and, thus, linearly independent of BMI-SDS). Continuous variables were z-transformed using the mean and standard deviation of the AN study group in accordance with the CLMI imputation model. FDR-q for follow-up BMI-SDS-adjusted RLMs: p-values were multiple testing adjusted using FDR across all follow-up RLMs; significant FDR-adjusted regression findings are flagged as: *, q<0.05.

[c]**Confirmatory RLM analysis for leptin concentrations ≥LOD (LOD=0.20µg/L)**: All AN participants with leptin concentrations <0.20µg/L were excluded to investigate trends of the association between log_{10}-leptin and amygdala nuclei volumes exclusively in cases where leptin concentration was measured with high accuracy and precision within the detection limits of the leptin assay. See table above for RLM formula with log_{10}-leptin as the predictor, applied to n=97 AN instead of n=142 AN. FDR-q: p-values were multiple testing adjusted using FDR across all confirmatory RLMs; significant FDR-adjusted regression findings are flagged as: *, q<0.05; **, q<0.01.

[d]**Exploratory RLMs for EDI-2 subscales/sub-scores "drive for thinness" and "body dissatisfaction"** separately (instead of "EDI-2 core" as the predictor) did not yield significant associations with amygdala nuclei volumes. **EDI-2 drive for thinness**: Accessory basal nucleus lh: t=0.09, p_{raw}=0.928; Accessory basal nucleus rh: t=-1.22, p_{raw}=0.225; Cortical nucleus lh: t=0.15, p_{raw}=0.878; Cortical nucleus rh: t=-1.88, p_{raw}=0.061; Corticoamygdaloid transition lh: t=0.53, p_{raw}=0.596; Corticoamygdaloid transition rh: t=-0.49, p_{raw}=0.626; Medial nucleus lh: t=0.17, p_{raw}=0.862. **EDI-2 body dissatisfaction**: Accessory basal nucleus lh: t=-0.42, p_{raw}=0.677; Accessory basal nucleus rh: t=-1.35, p_{raw}=0.181; Cortical nucleus lh: t=0.33, p_{raw}=0.744; Cortical nucleus rh: t=-1.07, p_{raw}=0.287; Corticoamygdaloid transition lh: t=0.39, p_{raw}=0.705; Corticoamygdaloid transition rh: t=-0.79, p_{raw}=0.436; Medial nucleus lh: t=0.35, p_{raw}=0.730.

SM 2.5 Robust linear regression analysis for associations between amygdala nuclei volumes and clinical measures in HC

Table S7. RLMs in the HC study group.

RLMs in HC (formula): Individual amygdala nuclei volumes (lh, rh) ~ clinical measure + $^{\perp}$poly(age^1, age^2) + eTIV

**Amygdala nuclei volumes** (same as in Table S6/AN study group)		**Clinical measures**	
		**Nutritional and neuroendocrine markers**	
		**BMI-SDS** (n=168)	**Log$_{10}$-leptin** (n=156)
Accessory basal nucleus lh	β	0.49	1.41
	95% CI	[-4.48, 5.46]	[-10.20, 13.02]
	t	0.19	0.24
	p	0.849	0.812
	η_p^2	<0.001	<0.001
Accessory basal nucleus rh	β	1.61	6.80
	95% CI	[-3.17, 6.40]	[-4.45, 18.05]
	t	0.66	1.18
	p	0.511	0.235
	η_p^2	0.003	0.009
Cortical nucleus lh	β	0.13	-0.16
	95% CI	[-0.62, 0.87]	[-1.91, 1.60]
	t	0.33	-0.18
	p	0.740	0.860
	η_p^2	<0.001	<0.001
Cortical nucleus rh	β	0.66	1.20
	95% CI	[-0.07, 1.38]	[-0.62, 3.01]
	t	1.78	1.29
	p	0.076	0.195
	η_p^2	0.019	0.011

Corticoamygdaloid transition lh	β	-0.41	-2.23
	95% CI	[-3.76, 2.93]	[-10.28, 5.82]
	t	-0.24	-0.54
	p	0.810	0.586
	η_p^2	<0.001	0.002
Corticoamygdaloid transition rh	β	1.55	4.52
	95% CI	[-2.13, 5.22]	[-4.58, 13.63]
	t	0.830	0.97
	p	0.412	0.328
	η_p^2	0.004	0.006
Medial nucleus lh	β	-0.37	-0.62
	95% CI	[-1.43, 0.68]	[-3.08, 1.85]
	t	-0.70	-0.49
	p	0.486	0.625
	η_p^2	0.003	0.002

Abbreviations: RLM, robust linear model; HC, healthy control participants; lh, left brain hemisphere; rh, right brain hemisphere; [1]poly(age^1, age^2), linear and quadratic orthogonal polynomials of age at date of research due to evidence for nonlinear age effects on amygdala volumes (Chen et al., 2016; Han et al., 2020; Kurth et al., 2019; Pomponio et al., 2020; Sämann et al., 2020; Vinke et al., 2018), orthogonalization to prevent multicollinearity (Chen et al., 2016); eTIV, estimated total intracranial volume; BMI-SDS, body mass index standard deviation score; log$_{10}$-leptin, logarithmically transformed (base 10) leptin concentration. RLMs in the HC study group (n_{total}=168, available n given in each column) for individual amygdala nuclei volumes (in alphabetical order, same selection of nuclei as in the AN study group, see Table S6), predicted by nutritional and neuroendocrine markers (BMI-SDS and log$_{10}$-leptin) and covarying for age at date of research (linear and quadratic orthogonal polynomials) and eTIV (each table cell represents an individual RLM, continuous independent variables were mean-centered using the mean of the HC study group). As model statistics, unstandardized regression coefficient of the predictor (β) and 95% confidence interval (CI), t-value (equals unstandardized β divided by its standard error), unadjusted p-value (p computed via robust Wald F-test), and effect size

estimate partial η^2 (Cohen, 1988) of the predictor are stated for each RLM. FDR-q: p-values were multiple testing adjusted using false discovery rate (FDR) (Benjamini & Hochberg, 1995) across all RLMs. No significant nominal, let alone FDR-adjusted regression findings emerged.

SM References

Amat, J. A., Bansal, R., Whiteman, R., Haggerty, R., Royal, J., & Peterson, B. S. (2008). Correlates of intellectual ability with morphology of the hippocampus and amygdala in healthy adults. *Brain and Cognition*, *66*(2), 105–114. https://doi.org/10.1016/j.bandc.2007.05.009

Annett, M. (1970). A classification of hand preference by association. *British Journal of Psychology*, *61*(3), 303–321. https://doi.org/10.1111/j.2044-8295.1970.tb01248.x

Annett, M. (1994). Handedness as a continuous variable with dextral shift: Sex, generation, and family handedness in subgroups of left- and right-handers. *Behavior Genetics*, *24*(1), 51–63. https://doi.org/10.1007/BF01067928

Baron, S., Courbebaisse, M., Lepicard, E. M., & Friedlander, G. (2015). Assessment of hydration status in a large population. *British Journal of Nutrition*, *113*(1), 147–158. https://doi.org/10.1017/S0007114514003213

Benjamini, Y., & Hochberg, Y. (1995). Controlling the False Discovery Rate: A Practical and Powerful Approach to Multiple Testing. *Journal of the Royal Statistical Society: Series B (Methodological)*, *57*(1), 289–300. https://doi.org/10.1111/j.2517-6161.1995.tb02031.x

Biller, A., Reuter, M., Patenaude, B., Homola, G. A., Breuer, F., Bendszus, M., & Bartsch, A. J. (2015). Responses of the Human Brain to Mild Dehydration and Rehydration Explored In Vivo by 1H-MR Imaging and Spectroscopy. *American Journal of Neuroradiology*, *36*(12), 2277–2284. https://doi.org/10.3174/ajnr.A4508

Boss, J., Mukherjee, B., Ferguson, K. K., Aker, A., Alshawabkeh, A. N., Cordero, J. F., Meeker, J. D., & Kim, S. (2019). Estimating Outcome-Exposure Associations when Exposure Biomarker Detection Limits vary Across Batches: *Epidemiology*, *30*(5), 746–755. https://doi.org/10.1097/EDE.0000000000001052

Boss, J., & Rix, A. (2020). *Lodi: Limit of Detection Imputation for Single-Pollutant Models. R package version 0.9.2*. https://CRAN.R-project.org/package=lodi

Chen, H., Zhao, B., Cao, G., Proges, E. C., O'Shea, A., Woods, A. J., & Cohen, R. A. (2016). Statistical Approaches for the Study of Cognitive and Brain Aging. *Frontiers in Aging Neuroscience*, *8*. https://doi.org/10.3389/fnagi.2016.00176

Cherbuin, N., Sachdev, P. S., & Anstey, K. J. (2011). Mixed handedness is associated with greater age-related decline in volumes of the hippocampus and amygdala: The PATH through life study. *Brain and Behavior*, *1*(2), 125–134. https://doi.org/10.1002/brb3.24

Cohen, J. (1960). A coefficient of agreement for nominal scales. *Educational and Psychological Measurement*, *20*(1), 37–46.

Cohen, J. (1988). *Statistical power analysis for the behavioral sciences (2nd ed.)*. Erlbaum.

Durazzo, T. C., Meyerhoff, D. J., Yoder, K. K., & Murray, D. E. (2017). Cigarette smoking is associated with amplified age-related volume loss in subcortical brain regions. *Drug and Alcohol Dependence*, *177*, 228–236. https://doi.org/10.1016/j.drugalcdep.2017.04.012

Dusi, N., Barlati, S., Vita, A., & Brambilla, P. (2015). Brain Structural Effects of Antidepressant Treatment in Major Depression. *Current Neuropharmacology*, *13*(4), 458–465. https://doi.org/10.2174/1570159X1304150831121909

Eddy, K. T., Lawson, E. A., Meade, C., Meenaghan, E., Horton, S. E., Misra, M., Klibanski, A., & Miller, K. K. (2015). Appetite Regulatory Hormones in Women With Anorexia Nervosa: Binge-Eating/Purging Versus Restricting Type. *The Journal of Clinical Psychiatry*, *76*(01), 19–24. https://doi.org/10.4088/JCP.13m08753

Ehrlich, S., Burghardt, R., Schneider, N., Broecker-Preuss, M., Weiss, D., Merle, J. V., Craciun, E. M., Pfeiffer, E., Mann, K., Lehmkuhl, U., & Hebebrand, J. (2009). The role of leptin and cortisol in hyperactivity in patients with acute and weight-recovered anorexia nervosa. *Progress in Neuro-Psychopharmacology and Biological Psychiatry*, *33*(4), 658–662. https://doi.org/10.1016/j.pnpbp.2009.03.007

Elbejjani, M., Auer, R., Jacobs, D. R., Haight, T., Davatzikos, C., Goff, D. C., Bryan, R. N., & Launer, L. J. (2019). Cigarette smoking and gray matter brain volumes in middle age

adults: The CARDIA Brain MRI sub-study. *Translational Psychiatry*, *9*(1), 78. https://doi.org/10.1038/s41398-019-0401-1

Etkin, A., & Wager, T. D. (2007). Functional Neuroimaging of Anxiety: A Meta-Analysis of Emotional Processing in PTSD, Social Anxiety Disorder, and Specific Phobia. *American Journal of Psychiatry*, *164*(10), 1476–1488. https://doi.org/10.1176/appi.ajp.2007.07030504

Fichter, M., & Quadflieg, N. (1999). *Strukturiertes Inventar für Anorektische und Bulimische Eßstörungen nach DSM-IV und ICD-10 (SIAB).* (1st ed.). Hogrefe.

Fichter, M., & Quadflieg, N. (2001). The structured interview for anorexic and bulimic disorders for DSM-IV and ICD-10 (SIAB-EX): Reliability and validity. *European Psychiatry*, *16*(1), 38–48. https://doi.org/10.1016/S0924-9338(00)00534-4

Fischl, B. (2004). Automatically Parcellating the Human Cerebral Cortex. *Cerebral Cortex*, *14*(1), 11–22. https://doi.org/10.1093/cercor/bhg087

Fischl, B., Salat, D. H., Busa, E., Albert, M., Dieterich, M., Haselgrove, C., van der Kouwe, A., Killiany, R., Kennedy, D., Klaveness, S., Montillo, A., Makris, N., Rosen, B., & Dale, A. M. (2002). Whole Brain Segmentation. *Neuron*, *33*(3), 341–355. https://doi.org/10.1016/S0896-6273(02)00569-X

Fox, J., & Weisberg, S. (2019). *car: An {R} Companion to Applied Regression*. Sage. https://socialsciences.mcmaster.ca/jfox/Books/Companion/

Hamilton, J. P., Siemer, M., & Gotlib, I. H. (2008). Amygdala volume in major depressive disorder: A meta-analysis of magnetic resonance imaging studies. *Molecular Psychiatry*, *13*(11), 993–1000. https://doi.org/10.1038/mp.2008.57

Han, L. K., Dinga, R., Hahn, T., Ching, C. R., Eyler, L. T., Aftanas, L., Aghajani, M., Aleman, A., Baune, B. T., Berger, K., Brak, I., Filho, G. B., Carballedo, A., Connolly, C. G., Couvy-Duchesne, B., Cullen, K. R., Dannlowski, U., Davey, C. G., Dima, D., … Schmaal, L. (2020). Brain aging in major depressive disorder: Results from the ENIGMA major depressive disorder working group. *Molecular Psychiatry*. https://doi.org/10.1038/s41380-020-0754-0

Hansen, B. B., & Klopfer, S. O. (2006). Optimal Full Matching and Related Designs via Network Flows. *Journal of Computational and Graphical Statistics*, *15*(3), 609–627. https://doi.org/10.1198/106186006X137047

Hao, Y., Bertolero, M., & Farah, M. J. (2022). Anger, Fear, and Sadness: Relations to Socioeconomic Status and the Amygdala. *Journal of Cognitive Neuroscience*, 1–11. https://doi.org/10.1162/jocn_a_01892

Hayano, F., Nakamura, M., Asami, T., Uehara, K., Yoshida, T., Roppongi, T., Otsuka, T., Inoue, T., & Hirayasu, Y. (2009). Smaller amygdala is associated with anxiety in patients with panic disorder: Smaller amygdala and anxiety in PD. *Psychiatry and Clinical Neurosciences*, *63*(3), 266–276. https://doi.org/10.1111/j.1440-1819.2009.01960.x

He, H., Wang, W., Crits-Christoph, P., Gallop, R., & Tang, W. (2017). *On the implication of structural zeros as independent variables in regression analysis: Applications to alcohol research*. 25.

Hebebrand, J., Muller, T. D., Holtkamp, K., & Herpertz-Dahlmann, B. (2007). The role of leptin in anorexia nervosa: Clinical implications. *Molecular Psychiatry*, *12*(1), 23–35. https://doi.org/10.1038/sj.mp.4001909

Ho, D., Imai, K., King, G., & Stuart, E. (2011). MatchIt: Nonparametric Preprocessing for Parametric Causal Inference. *Journal of Statistical Software*, *42*(8), 1–28.

Holtkamp, K., Herpertz-Dahlmann, B., Hebebrand, K., Mika, C., Kratzsch, J., & Hebebrand, J. (2006). Physical Activity and Restlessness Correlate with Leptin Levels in Patients with Adolescent Anorexia Nervosa. *Biological Psychiatry*, *60*(3), 311–313. https://doi.org/10.1016/j.biopsych.2005.11.001

Jassam, N., Luvai, A., Narayanan, D., Turnock, D., Lee, G., Earp, K., West, J., Day, A., Jeffery, J., Zouwail, S., El-Farhan, N., Dearman, R., Hayden, K., Willett, S., Osborne, J., & Barth, J. (2020). Albumin and calcium reference interval using healthy individuals and a data-mining approach. *Annals of Clinical Biochemistry: International*

Journal of Laboratory Medicine, *57*(5), 373–381. https://doi.org/10.1177/0004563220944204

Kong, X., Postema, M. C., Guadalupe, T., Kovel, C., Boedhoe, P. S. W., Hoogman, M., Mathias, S. R., Rooij, D., Schijven, D., Glahn, D. C., Medland, S. E., Jahanshad, N., Thomopoulos, S. I., Turner, J. A., Buitelaar, J., Erp, T. G. M., Franke, B., Fisher, S. E., Heuvel, O. A., … Francks, C. (2020). Mapping brain asymmetry in health and disease through the ENIGMA consortium. *Human Brain Mapping*, hbm.25033. https://doi.org/10.1002/hbm.25033

Kurth, F., Cherbuin, N., & Luders, E. (2019). Age but no sex effects on subareas of the amygdala. *Human Brain Mapping*, *40*(6), 1697–1704. https://doi.org/10.1002/hbm.24481

Landis, J. R., & Koch, G. G. (1977). An Application of Hierarchical Kappa-type Statistics in the Assessment of Majority Agreement among Multiple Observers. *Biometrics*, *33*(2), 363. https://doi.org/10.2307/2529786

Leon, S. J., Björck, Å., & Gander, W. (2013). Gram-Schmidt orthogonalization: 100 years and more. *Numerical Linear Algebra with Applications*, *20*(3), 492–532. https://doi.org/10.1002/nla.1839

Logue, M. W., van Rooij, S. J. H., Dennis, E. L., Davis, S. L., Hayes, J. P., Stevens, J. S., Densmore, M., Haswell, C. C., Ipser, J., Koch, S. B. J., Korgaonkar, M., Lebois, L. A. M., Peverill, M., Baker, J. T., Boedhoe, P. S. W., Frijling, J. L., Gruber, S. A., Harpaz-Rotem, I., Jahanshad, N., … Morey, R. A. (2018). Smaller Hippocampal Volume in Posttraumatic Stress Disorder: A Multisite ENIGMA-PGC Study: Subcortical Volumetry Results From Posttraumatic Stress Disorder Consortia. *Biological Psychiatry*, *83*(3), 244–253. https://doi.org/10.1016/j.biopsych.2017.09.006

Merz, E. C., Tottenham, N., & Noble, K. G. (2018). Socioeconomic Status, Amygdala Volume, and Internalizing Symptoms in Children and Adolescents. *Journal of Clinical Child & Adolescent Psychology*, *47*(2), 312–323. https://doi.org/10.1080/15374416.2017.1326122

Morey, R. A., Clarke, E. K., Haswell, C. C., Phillips, R. D., Clausen, A. N., Mufford, M. S., Saygin, Z., Wagner, H. R., LaBar, K. S., Brancu, M., Beckham, J. C., Calhoun, P. S., Dedert, E., Elbogen, E. B., Fairbank, J. A., Hurley, R. A., Kilts, J. D., Kimbrel, N. A., Kirby, A., … Yoash-Gantz, R. E. (2020). Amygdala Nuclei Volume and Shape in Military Veterans With Posttraumatic Stress Disorder. *Biological Psychiatry: Cognitive Neuroscience and Neuroimaging*, *5*(3), 281–290. https://doi.org/10.1016/j.bpsc.2019.11.016

Nave, G., Jung, W. H., Linnér, R. K., Kable, J. W., & Koellinger, P. D. (2019). *Are Bigger Brains Smarter? Evidence From a Large-Scale Preregistered Study*. *30*(1), 12. https://doi.org/10.1177/0956797618808470

Noble, K. G., Houston, S. M., Kan, E., & Sowell, E. R. (2012). Neural correlates of socioeconomic status in the developing human brain: Neural correlates of socioeconomic status. *Developmental Science*, *15*(4), 516–527. https://doi.org/10.1111/j.1467-7687.2012.01147.x

Patrick, K., Norman, G. J., Calfas, K. J., Sallis, J. F., Zabinski, M. F., Rupp, J., & Cella, J. (2004). Diet, Physical Activity, and Sedentary Behaviors as Risk Factors for Overweight in Adolescence. *Archives of Pediatrics & Adolescent Medicine*, *158*(4), 385. https://doi.org/10.1001/archpedi.158.4.385

Petermann, F., & Petermann, U. (2011). *Hamburg-Wechsler-Intelligenztest für Kinder–IV (HAWIK-IV)* (1st ed.). Pearson Assessment and Information GmbH.

Pietschnig, J., Penke, L., Wicherts, J. M., Zeiler, M., & Voracek, M. (2015). Meta-analysis of associations between human brain volume and intelligence differences: How strong are they and what do they mean? *Neuroscience & Biobehavioral Reviews*, *57*, 411–432. https://doi.org/10.1016/j.neubiorev.2015.09.017

Pomponio, R., Erus, G., Habes, M., Doshi, J., Srinivasan, D., Mamourian, E., Bashyam, V., Nasrallah, I. M., Satterthwaite, T. D., Fan, Y., Launer, L. J., Masters, C. L., Maruff, P., Zhuo, C., Völzke, H., Johnson, S. C., Fripp, J., Koutsouleris, N., Wolf, D. H., … Davatzikos, C. (2020). Harmonization of large MRI datasets for the analysis of brain

imaging patterns throughout the lifespan. *NeuroImage*, *208*, 116450. https://doi.org/10.1016/j.neuroimage.2019.116450

Popowski, L. A., Oppliger, R. A., Patrick Lambert, G., Johnson, R. F., Kim Johnson, A., & Gisolfi, C. V. (2001). Blood and urinary measures of hydration status during progressive acute dehydration: *Medicine and Science in Sports and Exercise*, 747–753. https://doi.org/10.1097/00005768-200105000-00011

Pujol, J., Soriano-Mas, C., Alonso, P., Cardoner, N., Menchón, J. M., Deus, J., & Vallejo, J. (2004). Mapping Structural Brain Alterations in Obsessive-Compulsive Disorder. *Archives of General Psychiatry*, *61*(7), 720. https://doi.org/10.1001/archpsyc.61.7.720

R Core Team. (2022). *R: A language and environment for statistical computing*. R Foundation for Statistical Computing. https://www.R-project.org/

Rubin, D. B. (1987). *Multiple Imputation for Nonresponse in Surveys*. John Wiley and Sons.

Sämann, P. G., Iglesias, J. E., Gutman, B., Grotegerd, D., Leenings, R., Flint, C., Dannlowski, U., Clarke-Rubright, E. K., Morey, R. A., Erp, T. G. M., Whelan, C. D., Han, L. K. M., Velzen, L. S., Cao, B., Augustinack, J. C., Thompson, P. M., Jahanshad, N., & Schmaal, L. (2020). FreeSurfer-based segmentation of hippocampal subfields: A review of methods and applications, with a novel quality control procedure for ENIGMA studies and other collaborative efforts. *Human Brain Mapping*, hbm.25326. https://doi.org/10.1002/hbm.25326

Saygin, Z. M., Kliemann, D., Iglesias, J. E., van der Kouwe, A. J. W., Boyd, E., Reuter, M., Stevens, A., Van Leemput, K., McKee, A., Frosch, M. P., Fischl, B., & Augustinack, J. C. (2017). High-resolution magnetic resonance imaging reveals nuclei of the human amygdala: Manual segmentation to automatic atlas. *NeuroImage*, *155*, 370–382. https://doi.org/10.1016/j.neuroimage.2017.04.046

Schorr, M., & Miller, K. K. (2017). The endocrine manifestations of anorexia nervosa: Mechanisms and management. *Nature Reviews Endocrinology*, *13*(3), 174–186. https://doi.org/10.1038/nrendo.2016.175

Stratmann, M., Konrad, C., Kugel, H., Krug, A., Schöning, S., Ohrmann, P., Uhlmann, C., Postert, C., Suslow, T., Heindel, W., Arolt, V., Kircher, T., & Dannlowski, U. (2014). Insular and Hippocampal Gray Matter Volume Reductions in Patients with Major Depressive Disorder. *PLoS ONE*, *9*(7), e102692. https://doi.org/10.1371/journal.pone.0102692

Streitbürger, D.-P., Möller, H. E., Tittgemeyer, M., Hund-Georgiadis, M., Schroeter, M. L., & Mueller, K. (2012). Investigating Structural Brain Changes of Dehydration Using Voxel-Based Morphometry. *PLoS ONE*, *7*(8), e44195. https://doi.org/10.1371/journal.pone.0044195

Szabo, C. A., Xiong, J., Lancaster, J. L., Rainey, L., & Fox, P. (2001). *Amygdalar and Hippocampal Volumetry in Control Participants: Differences Regarding Handedness*.

Szeszko, P. R., MacMillan, S., McMeniman, M., Lorch, E., Madden, R., Ivey, J., Banerjee, S. P., Moore, G. J., & Rosenberg, D. R. (2004). Amygdala Volume Reductions in Pediatric Patients with Obsessive–Compulsive Disorder Treated with Paroxetine: Preliminary Findings. *Neuropsychopharmacology*, *29*(4), 826–832. https://doi.org/10.1038/sj.npp.1300399

Tukey, J. W. (1977). *Exploratory Data Analysis*. Addison-Wesley.

van Buuren, S., & Groothuis-Oudshoorn, K. (2011). mice: Multivariate Imputation by Chained Equations in R. *Journal of Statistical Software*, *45*(3), 1–67. https://doi.org/10.18637/jss.v045.i03

van der Plas, E. A. A., Boes, A. D., Wemmie, J. A., Tranel, D., & Nopoulos, P. (2010). Amygdala volume correlates positively with fearfulness in normal healthy girls. *Social Cognitive and Affective Neuroscience*, *5*(4), 424–431. https://doi.org/10.1093/scan/nsq009

Venables, W. N., & Ripley, B. D. (2002). *MASS: Modern Applied Statistics with S*. Springer. http://www.stats.ox.ac.uk/pub/MASS4/

Vinke, E. J., de Groot, M., Venkatraghavan, V., Klein, S., Niessen, W. J., Ikram, M. A., & Vernooij, M. W. (2018). Trajectories of imaging markers in brain aging: The

Rotterdam Study. *Neurobiology of Aging*, *71*, 32–40. https://doi.org/10.1016/j.neurobiolaging.2018.07.001

von Aster, M., Neubauer, A., & Horn, R. (2006). *Wechsler Intelligenztest für Erwachsene (WIE). Deutschsprachige Bearbeitung und Adaptation des WAIS-III von David Wechsler*. Harcourt Test Services.

Wagner, A., Greer, P., Bailer, U. F., Frank, G. K., Henry, S. E., Putnam, K., Meltzer, C. C., Ziolko, S. K., Hoge, J., McConaha, C., & Kaye, W. H. (2006). Normal Brain Tissue Volumes after Long-Term Recovery in Anorexia and Bulimia Nervosa. *Biological Psychiatry*, *59*(3), 291–293. https://doi.org/10.1016/j.biopsych.2005.06.014

Watkins, K. E. (2001). Structural Asymmetries in the Human Brain: A Voxel-based Statistical Analysis of 142 MRI Scans. *Cerebral Cortex*, *11*(9), 868–877. https://doi.org/10.1093/cercor/11.9.868

Wickham, H. (2016). *ggplot2: Elegant Graphics for Data Analysis*. Springer-Verlag New York. https://ggplot2.tidyverse.org

Wilkinson, G. N., & Rogers, C. E. (1973). Symbolic Description of Factorial Models for Analysis of Variance. *Applied Statistics*, *22*(3), 392. https://doi.org/10.2307/2346786

Young, K. D., Friedman, E. S., Collier, A., Berman, S. R., Feldmiller, J., Haggerty, A. E., Thase, M. E., & Siegle, G. J. (2020). Response to SSRI intervention and amygdala activity during self-referential processing in major depressive disorder. *NeuroImage: Clinical*, *28*, 102388. https://doi.org/10.1016/j.nicl.2020.102388
